# Differing Content and Language Based on Poster-Patient Relationships on the Chinese Social Media Platform Weibo: Text Classification, Sentiment Analysis, and Topic Modeling of Posts on Breast Cancer

**DOI:** 10.2196/51332

**Published:** 2024-05-09

**Authors:** Zhouqing Zhang, Kongmeng Liew, Roeline Kuijer, Wan Jou She, Shuntaro Yada, Shoko Wakamiya, Eiji Aramaki

**Affiliations:** 1 Graduate School of Science and Technology Nara Institute of Science and Technology Ikoma Japan; 2 School of Psychology, Speech and Hearing University of Canterbury Christchurch New Zealand; 3 Department of Information Science Kyoto Institute of Technology Kyoto Japan

**Keywords:** cancer, social media, text classification, topic modeling, sentiment analysis, Weibo

## Abstract

**Background:**

Breast cancer affects the lives of not only those diagnosed but also the people around them. Many of those affected share their experiences on social media. However, these narratives may differ according to who the poster is and what their relationship with the patient is; a patient posting about their experiences may post different content from someone whose friends or family has breast cancer. Weibo is 1 of the most popular social media platforms in China, and breast cancer–related posts are frequently found there.

**Objective:**

With the goal of understanding the different experiences of those affected by breast cancer in China, we aimed to explore how content and language used in relevant posts differ according to who the poster is and what their relationship with the patient is and whether there are differences in emotional expression and topic content if the patient is the poster themselves or a friend, family member, relative, or acquaintance.

**Methods:**

We used Weibo as a resource to examine how posts differ according to the different poster-patient relationships. We collected a total of 10,322 relevant Weibo posts. Using a 2-step analysis method, we fine-tuned 2 Chinese Robustly Optimized Bidirectional Encoder Representations from Transformers (BERT) Pretraining Approach models on this data set with annotated poster-patient relationships. These models were lined in sequence, first a binary classifier (no_patient or patient) and then a multiclass classifier (post_user, family_members, friends_relatives, acquaintances, heard_relation), to classify poster-patient relationships. Next, we used the Linguistic Inquiry and Word Count lexicon to conduct sentiment analysis from 5 emotion categories (positive and negative emotions, anger, sadness, and anxiety), followed by topic modeling (BERTopic).

**Results:**

Our binary model (*F*_1_-score=0.92) and multiclass model (*F*_1_-score=0.83) were largely able to classify poster-patient relationships accurately. Subsequent sentiment analysis showed significant differences in emotion categories across all poster-patient relationships. Notably, negative emotions and anger were higher for the “no_patient” class, but sadness and anxiety were higher for the “family_members” class. Focusing on the top 30 topics, we also noted that topics on fears and anger toward cancer were higher in the “no_patient” class, but topics on cancer treatment were higher in the “family_members” class.

**Conclusions:**

Chinese users post different types of content, depending on the poster- poster-patient relationships. If the patient is family, posts are sadder and more anxious but also contain more content on treatments. However, if no patient is detected, posts show higher levels of anger. We think that these may stem from rants from posters, which may help with emotion regulation and gathering social support.

## Introduction

### Background

Breast cancer is 1 of the most common forms of cancer, with an estimated 2 billion people being affected worldwide in 2020 (according to statistics released by the World Health Organization [WHO]), and is consequently a disease familiar to many people. It is a chronic disease with a high mortality rate, which poses a serious threat to human life [1]. For this reason, breast cancer is often viewed negatively, and new diagnoses often trigger sadness, fear, and even psychopathological comorbidities, such as depression [2]. In recent decades, the number of new diagnoses has continued to rise, despite important improvements in medical technologies worldwide [1]. In China, more than 400,000 people were diagnosed with breast cancer in 2020, with approximately 100,000 deaths (according to WHO) [1]. Behind these diagnoses are numerous stories emerging from the experiences of patients or the people around them who are closely intertwined [3]. Therefore, it is not unusual for one to come across discussions on breast cancer in daily life—be it learning about the diagnosis of a loved one or acquaintance or coming across news on a celebrity with breast cancer or even struggling to accept the diagnosis of a close relative. Therefore, a lot of these breast cancer–related narratives take place on social media–lived experiences of people who may have been diagnosed with or who know of someone struggling with breast cancer.

Social media is indispensable in the daily life of billions worldwide; almost everyone is a user of a social media platform [4]. On these platforms, people can share snippets of their lives with other people around them, which double as autobiographical records of their life events. As a social tool, one can smoothly interact and communicate with one’s friends and family over the internet, be it synchronously or asynchronously [5,6]. Such activity leaves digital traces all over the internet, and researchers have since begun using social media posts as resources for uncovering social phenomena [5]. Particularly in the medical field, social media analyses have also been used to great effect, for example, in examining and predicting the epidemiological spread of infectious diseases, such as seasonal influenza and COVID-19 [7,8]. Recently, researchers have also analyzed social media to learn about the perspectives and needs of patients with certain diseases. For example, Kamba et al [9] analyzed a Japanese social media forum (Yahoo Japan) for posts relating to breast cancer and found that the most frequently mentioned concerns pertain to symptoms, screening, and lack of knowledge, to name a few (see also Refs. [10,11]).

However, much of this research has been conducted on Western social media platforms, such as Twitter and Reddit, which have limited penetration in the Chinese market. Chinese internet users have their own social media ecosystems and platforms: Sina Weibo is 1 of the most widely used and popular social platforms in China and has been called by some as the “Chinese version of Twitter” [12]. Given our research interest in Chinese social media users, we focused our paper specifically on Weibo. As a widely used platform, the number of monthly active users reached 511 million in 2020; Weibo is known by almost everyone in China [13], and posts are known to reflect the diversity of opinions and perspectives by everyday Chinese [14]. Often, users discuss and post about all kinds of topics on Weibo, including topics pertaining to breast cancer. With the large number of users and the diversity of content, Weibo data appear to be a valuable corpus for research on Chinese perspectives from the bottom-up.

### Sentiment Analysis on Social Media

To accommodate the large volume of data on the internet, conventional methods, such as qualitative coding, may be too time-consuming and costly. Therefore, modern sociological researchers frequently use computational methods, such as sentiment analysis and topic modeling, to analyze the data. Originating from the field of natural language processing (NLP), sentiment analysis is optimized to deal with the detection and classification of sentiments in (a large number of) texts. By using sentiment analysis, we can infer whether a given text has a positive, negative, or more fine-grained emotional orientation in a given context [15]. In studying social media, researchers analyze the data on social media to obtain public perceptions on a specific topic in contribution to the study and advancement of society [16]. Some researchers have also applied sentiment analysis to measure customers’ needs from their social media posts, thereby obtaining unique insight to improve a brand’s products or services [15]. Researchers have also applied sentiment analysis on social media to predict mental health issues, for example, Wang et al [17] used sentiment analysis to detect users with depression on social networking services.

Regarding breast cancer, sentiment analysis may play a more important role in exploring the patients’ psychological state, such as their perceptions, cognitions, and emotions [18]. Through analyses of tweet sentiments, previous research has confirmed that patients with breast cancer have different polarities (valence) of emotional expression for topics related to breast cancer [19]. For example, support seeking and treatments are associated with positive sentiment, but health care and insurance are associated with negative sentiment. Moreover, posters may not necessarily be patients themselves posting about their experiences or concerns but could be posting about a loved one, a relative, or an acquaintance with breast cancer. Accordingly, posters’ emotional expressions on social media may not only display differences in sentiment, depending on their specified content or aspects (eg, treatment stage or success), but also show differences, depending on their relationship with the patient [20] or if the posters themselves are the patients. In this paper, we define this as the “poster-patient relationship.” Therefore, in studying the usage of social media for emotional expression in the context of breast cancer, we propose the necessity to distinguish the poster-patient relationships for each post—whether posts originate from patients themselves or from their friends and relatives or other people.

### The Research

Before examining emotional expressions and sentiment, we intended to discern the relationships between poster and patient through the post. Due to the large volume of data, we turned to machine learning for this task. “Machine learning” is the term used to describe both the academic discipline and the collection of techniques that allow computers to undertake complex tasks, and recent advances in machine learning have driven advances in the development of NLP and artificial intelligence (AI) [21]. In NLP, the past 5 years have seen rapid advances in the transformer-based framework, resulting in cutting-edge pretrained language models, such as Bidirectional Encoder Representations from Transformers (BERT) [22], Robustly Optimized BERT Pretraining Approach (RoBERTa) [23], and Generative Pretrained Transformer (GPT)-3 [24], which have greatly improved the effectiveness of downstream tasks (eg, text classification), opening up new avenues for researchers to study society and language [25].

Our aim was to study how users on the Chinese social media platform Weibo post about breast cancer–related topics on social media. Although we took a hypothesis-blind, exploratory approach to data analysis, we focused our discussion on topics surrounding the issue of emotional expression by examining differences in emotional expression, depending on poster-patient relationships. In step 1, we collected data from Weibo and determined poster-patient relationships through 2 stages of classification: first, we identified whether a post references a patient with breast cancer (as opposed to posts that mention breast cancer without naming a specific patient), followed by the poster-patient relationship classification that determined the relationship between the mentioned patient and the author of the post (poster). Ultimately, these 2 stages in step 1 constituted a single classification pipeline to identify poster-patient relationships: whether the post authors are themselves the patients or (1) a family member (*family_members*); (2) a friend or relative (*friends_relatives*); (3) an acquaintance (*acquaintances*); (4) from a parasocial relationship, such as a celebrity or public figure (*heard_relation*); or (5) no patient mentioned (*no_patient*). In step 2, we used the LIWC-based dictionary to count the word frequency for each post, with 5 emotional categories (sadness, anger, anxiety, positive, and negative), thereby expanding our target beyond just positive and negative sentiments. Despite the lack of discreet positive emotion categories in the LIWC dictionary, we chose it because it is 1 of the most widely used and accessible sentiment dictionaries in psycholinguistic research. Next, we used topic modeling to further examine the main topics discussed between each class and how these topics differ across classes. This will allow us to see how social media narratives for patients and posters differ, while shedding light on possible implications for emotional expression via social media.

## Methods

### Ethical Considerations

As all data used in this study are publicly available and no personal identifiers were obtained, our study was exempt from institutional ethics review. Where applicable, all posts included in this analysis have been paraphrased so that they cannot be traced back to the user. No identifying information (eg, usernames, IDs, or pictures) are included in the main manuscript or in the supplementary material.

### Step 1: Poster-Patient Relationship Classification

#### Data Collection

Since Sina Weibo does not maintain a public application programming interface (API), we used a previously constructed web crawler to request publicly available Weibo posts. Our web crawler simulates a user visiting Weibo’s official website and searches for relevant posts (see the next paragraph for the search procedure). Through this approach, each web search request can obtain up to 50 posts before reinitiating a new search request to retrieve a new set of posts. In our crawler, we were able to set adjustable parameters to specify keywords, the publishing date, location, and interval times between 2 search requests.

We conducted 2 searches with different queries: “breast cancer (‘乳腺癌’)” and “sadness (‘悲伤’)”, as well as “breast cancer (‘乳腺癌’)” and “record (‘记录’)” in Chinese, from January 1, 2018, to December 31, 2021. For both queries, the interval time was set to 15 seconds and the location was unspecified, meaning that we searched for posts from across China. Finally, for the 2 searches with different queries, we obtained 160,182, and 144,125 posts, respectively. For each post, we additionally obtained the user id, username, user type, publish time, post text, location, number of comments, likes, and reposts, which were removed before commencement of analyses.

Next, for the data-cleaning phase, we combined the search results of the 2 queries into a single data set. Duplicate posts were removed through string matching, and obvious advertisements and irrelevant posts were removed by manually checking the data set. This was to ensure the posts were related to narrative accounts pertaining to breast cancer. Finally, this resulted in a cleaned data set containing relevant breast cancer–related narratives from individual users, for a total of 10,322 posts.

#### Poster-Patient Relationship Classification Criteria

First, we set up 6 categories based on the relationship of the mentioned patient and the author of the post: “post_user,” where the authors are themselves the patients (coded as 0); “family_members,” where the authors mention a family member (eg, parent) as the patient (coded as 1); “friends_relatives,” where a friend or nonimmediate relative (eg, cousins, aunt) is the patient (coded as 2); “acquaintances,” where a colleague or neighbor (social relationships) is the patient (coded as 3); “heard_relation,” where the author may be posting about a celebrity or a famous patient with cancer (coded as 4); and “no_patient,” where breast cancer is mentioned generally without being associated with a specific person (coded as 5).

#### Data Annotation

We randomly portioned 3000 (29.1%) of the 10,322 posts for manual annotation based on the classification criteria, with each data point (post) assigned a label from the 6 aforementioned categories. In the process of labeling, first we determined whether there was a patient in the post (binary classification task), and then we determined whether the poster-patient relationship could be inferred and labeled according to the prespecified classification criteria (multiclass classification task). All data labeling was performed by 1 of the authors who is a native Chinese speaker. See Table 1 for the annotation proportions, and Table S1 in Multimedia Appendix 1 for examples of annotated posts.

To verify that our annotations were objectively labeled and free of subjective bias, we randomly selected 600 (20%) of the 3000 annotated posts, and these were reannotated in the same procedure by another native Chinese annotator who was not part of the research team. Across the 6 categories, the interannotator agreement was good (Cohen κ=0.67) [26], and the original annotations were used to train the classification model.

**Table 1:** Distribution of annotated posts.

	Posts, n
No_patient	1089
Heard_relation	509
Family_members	443
Acquaintances	356
Post_user	338
Friends_relatives	265

#### Data Preprocessing

In our study, we chose the pretrained Chinese-RoBERTa-wwm-ext (Chinese RoBERTa) [27] model as our classification model. The Chinese RoBERTa is a large language transformer model based on the RoBERTa architecture [23], trained on a large corpus of the in house–collected extended data containing an encyclopedia, news articles, and web forums, which has 5.4 billion words and is over 10 times bigger than the Chinese Wikipedia [27], and is frequently used for Chinese NLP tasks. To improve the accuracy of the multiclass text classification, we decomposed the classification task over 2 stages (see Ref. [28]): a binary classification task to determine whether a patient was mentioned, followed by a multiclass classifier on posts where a patient was mentioned in order to identify the poster-patient relationship.

The pretrained language model (Chinese RoBERTa) has a limited input character length of 512, and 522 posts in our data set were longer than this character length limit. As such, we used automated text summarization to condense the text length to within 512 characters for these 522 posts using SnowNLP, a Python library that can perform Chinese word segmentation, part-of-speech tagging, sentiment analysis, text categorization, *pinyin* conversion, traditional simplification, text keyword extraction, text summarization, sentence segmenting, and text similarity estimation [29]. The SnowNLP tool segments posts by sentence and using the TextRank algorithm [30] calculates the weight of each sentence in the post according to the extent to which the content of the sentence represents the content of the text. Finally, all the small units are sorted in reverse order according to their weight scores. When implementing this tool, by setting a number parameter, the corresponding number of sentences is output accordingly, resulting in summarized texts. In Multimedia Appendix 2, we included some examples of automatic summarization.

#### Classifier Training

Following annotation and data preprocessing, 2 classifiers were constructed for this study in a 2-stage process. In the first stage, a binary classification model was trained to identify whether a patient is mentioned. This was followed by training a multiclass classification model to identify the poster-patient relationship for each post where a patient was mentioned in 1 of 5 classes: post_user, family_members, friends_relatives, acquaintances, and heard_relation. This resulted in a total of 6 classes corresponding to the annotations, with the inclusion of the “no_patient” class from the earlier binary classification model. In constructing the 2 classifiers, we specified the task of the RoBERTa model as classification. We monitored the training performance for each epoch through cross-entropy loss. Fine-tuning was implemented under the Pytorch framework, where we used the Amda Optimizer to optimize and update model parameters for training purposes. For testing, sklearn metrics were used to evaluate the binary classification and multiclass classification. In addition, 2400 (80%) of the 3000 annotated posts were used to train the model, and the main parameters for the model training were as follows: batch size=16, learning rate=1.0 × 10^–5^, and training epochs=5. We used 600 (20%) posts to test the fine-tuned model.

In the second stage, we removed the “no_patient” class from the annotated data. In total, 1515 (50.5%) posts were used to fine-tune the Chinese RoBERTa model. The main parameters were similar to the binary classifier, with batch size=16, learning rate=1.0 × 10^–5^, and training epochs=5. For validation, we used 396 (13.2%) posts to test the trained model.

### Step 2: Examining Differences in Emotional Expression

#### Analysis 1: Sentiment Analysis Based on the LIWC

The LIWC program is a text analysis program that calculates the degree of use for different categories of words across a wide array of texts [31]. This tool was originally developed in English, but researchers have since produced a Chinese version of the LIWC dictionary based on the same criteria [32]. We used an open source Python package to access the Chinese LIWC dictionary. The LIWC dictionary has proved extremely useful in a number of different disciplines and has had a large impact on our understanding of how lexical elements related to cognition, affect, and personal concerns can be used to better understand human behavior [33].

In our study, we focused on the emotion categories to implement the sentiment analysis in our corpus of Weibo posts. We used the LIWC program and its Chinese dictionary to examine 5 emotion categories available in the Chinese LIWC dictionary: positive emotions, negative emotions, sadness, anger, and anxiety. The LIWC dictionary operates by counting the number of terms in each post that corresponds to its internal dictionary for each emotion category, and outputs a score representing the ratio of relevant terms to all identified terms in the post. We then conducted Kruskal-Wallis tests to determine whether positive emotion terms, negative emotion terms, anxiety terms, sadness terms, and anger terms significantly differed between each poster-patient relationship class. If there was a significant effect of the emotion category, we conducted post hoc Dwass-Steel-Critchlow-Fligner (DSCF) pairwise comparisons to compare differences between specific categories.

In this paper, our data are in Chinese, so we had to tokenize our data. We used Jieba for tokenization, which is 1 of the most popular Chinese tokenization tools in NLP [34]. To clean out the noise, we excluded more than 2000 stop words, which were collected from an open source Chinese dictionary of stop words.

#### Analysis 2: Topic Modeling

Making sense of a large unstructured corpus through qualitative means is difficult. Therefore, we used topic modeling to better assist us in interpreting data. Topic modeling is a widely used approach to extract common, recurring themes from large amounts of text data through identification and clustering of repeated patterns in words and sentences. In this paper, we adopted the open source BERTopic algorithm [35] to achieve this. BERTopic leverages transformers and class-based term frequency–inverse document frequency (c-TF-IDF) to create dense clusters of words, allowing for easily interpretable topics, while keeping important words in the topic descriptions [35]. Past research [36] has also found that BERTopic-based topic modeling generally yields more theoretically interpretable results than other forms of topic modeling (eg, latent Dirichlet allocation or Top2Vec). As the BERTopic algorithm only assigns 1 topic to every document (post), we were able to compute topics per class, which allowed uniform comparison of topic distribution for every class (poster-patient relationships), enabling us to observe general trends: which topics are more frequently observed in which class of poster-patient relationship. As long texts are more suitable for modeling and there is no limit to the length of input sentences, during the topic modeling, we replaced the summarized sentences with the original ones. For identified topics, we deliberated on the schema associated with as many words in the topic as possible. Note that this process is largely subjective, so we encourage readers to additionally reference the words contained in each topic, rather than relying solely on the authors’ labels.

In this paper, our data are in Chinese and because the BERTopic model is based on the clustering of individual words to implement topic modeling; therefore, in the process of topic modeling, similar to the sentiment analysis, we needed to tokenize our Chinese data. We again used Jieba for tokenization [34]. To obtain meaningful entities from the topic models, we excluded more than 2000 stop words, which were collected from an open source Chinese dictionary of stop words.

## Results

### Step 1: Poster-Patient Relationship Classification

#### Binary Classifier

This model was trained to distinguish each post as either mentioning (“patient” class) or not mentioning (“no_patient” class) a patient. We merged the “post_user,” “family_members,” “friends_relatives,” “acquaintances,” and “heard_relation” classes into a superordinate “patient” class. The model achieved a high *F*_1_-score (see Table 2).

**Table 2:** Binary classifier’s metrics report.

Class	Precision	Recall	*F*_1_-score	Support
no_patient	0.90	0.90	0.90	204
patient	0.95	0.95	0.95	396
Macro average	0.92	0.92	0.92	600

#### Multiclass Classifier

Next, we constructed a multiclass classifier to focus on patient classification: “post_user,” “family_members,” “friends_relatives,” “acquaintances,” and “heard_relation.” Results are reported in Table 3.

**Table 3:** Multiclass classifier’s metric report.

Class	Precision	Recall	*F*_1_-score	Support
acquaintances	0.76	0.67	0.71	75
heard_relation	0.83	0.83	0.83	102
famliy_members	0.93	0.90	0.91	86
post_user	0.86	0.91	0.89	82
friends_relatives	0.74	0.84	0.79	51
Macro average	0.82	0.83	0.83	396

#### Post Classification

After excluding the annotated data, we were left with 7322 (70.9%) of the 10,322 data points (posts). These posts then underwent the 2-stage classification process. The first stage included a binary classifier to determine whether patient information was identifiable from the post (patient and no_patient), and if a patient was detected, the post then passed to the second stage. This included a multiclass classifier to classify the relationship between the patient and the Weibo poster. In the first stage, 4494 (61.4%) posts were classified as having a patient and 2828 (38.6%) posts as having no patient. Of the former, the relation classifications were as follows (Table 4): the patient was identified as a friend or relative (friends_relatives; n=667, 14.8%), as the poster (post_user; n=705, 15.7%), as an acquaintance (acquaintances; n=781, 17.4%), as a family member (family_members; n=961, 21.4%), and as someone they had only heard about (heard_relation; n=1380, 30.7%).

As Tables 1 and 4 show, the rankings of categories by the number of relevant posts were similar regardless of whether the data were manually labeled or predicted by our classifier. The ranking list was no_patient > heard_relation > family_members > acquaintances > post_user > friends_relatives. We noted that the “no_patient” class that did not mention a specific patient was the majority class, which accounted for one-third of the total number of posts (n=2828, 38.6%). We think that posters use the target words (“breast cancer”) to share some personal thoughts, not necessarily about specific instances of breast cancer or for a targeted patient. Alternatively, they may feel no need to talk about the patient due to the content and style of the post. Except for this class, the distribution of the other poster-patient relationship classes was relatively balanced in the data set.

**Table 4:** Distribution of predicted posts.

	Posts, n
No_patient	2828
Heard_relation	1380
Family_members	961
Acquaintances	781
Post_user	705
Friends_relatives	667

### Step 2: Examining Differences in Emotional Expressions

#### Sentiment Analysis

For subsequent analyses, our aim was to maximize the information we could extract from the data, so manual annotations were combined with the machine-learned predictions for a total of 10,322 posts. We applied the LIWC and the matched Chinese dictionary to count the emotion-related words for each tokenized post. We mainly focused on positive emotion, negative emotion, sadness, anger, and anxiety categories. We calculated the ratio of each emotion category in each post (number of emotion words/number of all tokens). To visualize broad emotional differences among the classified poster-patient relationship classes, we plotted the mean scores for 6 identity categories in each of the 5 emotion categories.

For positive emotions, the “friends_relatives” class had a relatively higher value than the other 5 classes (Table 5). For negative emotions, the “no_patient” class had a relatively higher value than the other 5 classes. For angry terms, the “no_patient” class had a significantly higher value than the other 5 classes, which had almost the same values. For anxiety terms, the “family_members,” “no_patient,” and “post_user” classes had a higher value than the other 3 classes; the “heard_relation” class had the lowest value. For sadness terms, the “family_members,” “no_patient,” and “post_user” classes had a relatively higher value than the other 3 classes.

**Table 5:** Emotion distribution for each class in the 5 emotion categories (positive emotions, negative emotions, anger, anxiety, and sadness).

			Mean ratio of each emotion category in each post^a^
**Positive emotions**			
	no_patient	0.05567	
	heard_relation	0.05785	
	family_members	0.05469	
	acquaintances	0.06581	
	post_user	0.05382	
	friends_relatives	0.07490	
**Negative emotions**			
	no_patient	0.11920	
	heard_relation	0.09202	
	family_members	0.09933	
	acquaintances	0.09118	
	post_user	0.09759	
	friends_relatives	0.09386	
**Anger**			
	no_patient	0.01020	
	heard_relation	0.00490	
	family_members	0.00467	
	acquaintances	0.00479	
	post_user	0.00469	
	friends_relatives	0.00489	
**Anxiety**			
	no_patient	0.00699	
	heard_relation	0.00389	
	family_members	0.00674	
	acquaintances	0.00465	
	post_user	0.00595	
	friends_relatives	0.00430	
**Sadness**			
	no_patient	0.01094	
	heard_relation	0.00894	
	family_members	0.01107	
	acquaintances	0.00845	
	post_user	0.01110	
	friends_relatives	0.00928	

^a^Number of emotion words/number of all tokens.

Next, we statistically examined differences in emotions across poster-patient relationships. Kruskal-Wallis tests showed significant effects for positive emotions (posemo: *χ*^2^_5_=185.9, *P*<.001), negative emotions (negemo; *χ*^2^_5_=156.8, *P*<.001), anxiety (anx; *χ*^2^_5_=50.6, *P*<.001), anger (anger; *χ*^2^_5_=38.2, *P*<.001), and sadness (sad; *χ*^2^_5_=56.8, *P*<.001). This suggests that for all emotion categories, significant effects were detected across the 6 poster-patient relationship classes. Table 6 reports the post hoc DSCF pairwise comparisons.

Although there were a number of significant effects, here we comment primarily on consistent patterns of results that may be indicative of broader trends in Weibo users with respect to the emotional language used when posting about breast cancer. We noticed that the “friends_relatives” class had significantly higher positive emotions than all other poster-patient relationship classes, and this was followed closely by the “acquaintances” class, which had higher positive emotions than the other remaining poster-patient relationship classes.

**Table 6:** Pairwise comparisons for the 5 emotion categories.

Class 1	Class 2	Positive emotions			Negative emotions			Anxiety			Anger			Sadness	
		W^a^	*P* value	W		*P* value	W		*P* value	W		*P* value	W		*P* value
acquaintances	family_members	–7.87	<.001^b^	3.94		.06	7.55		<.001^b^	1.05		.98	6.42		<.001^b^
acquaintances	friends_relative	5.67	<.001^b^	1.87		.77	1.29		.94	1.75		.82	3.02		.27
acquaintances	heard_relation	–6.75	<.001^b^	0.64		0.99	0.91		.98	1.81		.79	2.47		.50
acquaintances	no_patient	–10.73	<.001^b^	12.13		<.001^b^	2.65		.42	6.38		<.001^b^	–0.46		.99
acquaintances	post_user	–8.49	<.001^b^	3.13		.23	5.15		.004^b^	1.74		.82	5.16		.004^b^
family_members	friends_relatives	13.42	<.001^b^	–1.83		.79	–5.90		<.001^b^	0.86		.99	–3.01		.27
family_members	heard_relation	1.78	.81	–3.96		.06	–7.94		<.001^b^	0.81		.99	–4.81		.01^b^
family_members	no_patient	–2.62	.43	8.63		<.001^b^	–6.92		<.001^b^	5.71		<.001^b^	–8.88		<.001^b^
family_members	post_user	–1.08	.97	–0.61		.99	–2.13		.66	0.86		.99	–0.87		.99
friends_relative	heard_relation	–12.65	<.001^b^	–1.54		.89	–0.57		.99	–0.21		.99	–1.04		.98
friends_relative	no_patient	–16.21	<.001^b^	9.41		<.001^b^	0.94		.98	4.01		.05	–4.23		.03^b^
friends_relative	post_user	–13.68	<.001^b^	1.19		.96	3.69		.09	–0.07		.99	2.02		.71
heard_relation	no_patient	–5.15	.004^b^	14.14		<.001^b^	2.01		.72	5.55		.001^b^	–4.14		.04^b^
heard_relation	post_user	–2.76	.37	2.98		.28	5.02		.005^b^	0.12		.99	3.47		.14
no_patient	post_user	1.29	.94	–8.31		<.001^b^	3.76		.08	–4.23		.03^b^	6.98		<.001^b^

^a^Standardized Wilcoxon statistic from Dwass-Steel-Critchlow-Fligner (DSCF) pairwise comparisons.

^b^Significant *P* values.

In addition, we found that “no_patient” posts had consistently higher negative emotions than the posts in all other poster-patient relationship classes, but no strong and consistent pattern of difference was observed between other poster-patient relationship classes. This pattern was mirrored strongly in the anger emotion category, suggesting that “no_patient” posts were higher on anger compared to posts in the other poster-patient relationship classes. As “negative emotions” is a broad emotion category containing many other negative emotion words in its dictionary, we think that strong differences observed in anger could be driving the significant difference found in the negative emotions category.

Furthermore, we noticed that with the exception of the “post_user” class, the “family_members” class was generally significantly higher in anxiety than the “acquaintances,” “friends_relatives,” “no_patient,” and “heard_relation” poster-patient relationship classes and higher in sadness than the “acquaintances,” “no_patient,” and “heard_relation” poster-patient relationship classes.

#### Clustered Topics

To gain an overview of why some poster-patient relationship classes were consistently higher in some emotions than other classes, we turned to topic modeling. Using the *topics per class* function of the BERTopic model, we aimed to compare topical relationships that mirrored some of the identified effects from the sentiment analysis.

We initially found that 139 topics were automatically generated from BERTopic, but this included several topics of low significance, where post counts numbered less than 50. As we wanted to focus on topics of greater relevance, we narrowed our analysis to include only the top 30 (21.6%) topics by topic prevalence across the entire data set, which was sufficient to cover more than 6000 (58.1%) posts. In Table 7 and in Table S2 in Multimedia Appendix 3, we list the top 30 topics with top 30 representative terms and provide a summarized theme for each topic. These are represented by an ID, which represents the ranked prevalence of each topic, while the topic number represents the topic labels assigned for the initial generation. We also visualized the distribution of (poster-patient relationship) classes per topic, which was used to identify topics that were more prevalent in a particular class for the analysis. These visualizations are available in our GitHub repository [37].

**Table 7:** Top 30 terms of top 30 topics from topic modeling.

ID	Topic number	Label	Top 30 representative words (Chinese)	Top 30 representative words (translated into English)
0	0	Anger	生气,脾气,气死我了,情绪,真的	angry, temper, I’m angry, emotions, really
1	1	Laments	去世,家里,回来,生活,记得	passed away, at home, come back, life, remember
2	3	Symptoms	乳腺,乳房,肿块,增生,结节,	breast, breast, lump, hyperplasia, node
3	4	Hospital stays	医生,病人,主任,医院,手术	doctor, patient, director, hospital, surgery
4	7	Hope and prayers	希望,幸福,生活,人生,幸运	hope, happiness, life, life, lucky
5	6	Hospitalization	手术,医院,化疗,住院,医生	surgery, hospital, chemotherapy, hospitalization, doctor
6	8	Lamenting hospitalization	病房,医院,病人,恐惧,患者	ward, hospital, patient, fear, patient
7	2	Dreams and nightmares	梦里,梦见,梦到,昨晚,做梦	dream, dreaming, dreaming, last night, dreaming
8	10	Diagnosis	一年,手术,去年,确诊,希望	a year, surgery, last year, diagnosed, hope
9	5	Chinese dramas	刘静,女主,男主,欢喜,英子	Liu Jing, heroine, hero, cheerful, Yingzi
10	13	School	老师,学生,家长,班主任,上课	teacher, student, parent, classroom, lesson
11	20	Friends	朋友,闺蜜,离婚,聊天,命理	friend, bestie, divorce, chat, numerology
12	18	Sleep-wake cycles	熬夜,睡觉,晚上,睡不着,睡着	stay up, sleep, night, sleepless, sleep
13	12	Passing	去世,消息,难过,死者,刚刚	passed away, news, sad, deceased, just
14	26	Treatment processes	放疗,化疗,结束,治疗,转移	radiotherapy, chemotherapy, end, treatment, metastasis
15	33	Treatment effects	治愈,治疗,方案,效果,患者	cure, treatment, protocol, effect, patient
16	113	Appeal to emotion	开心,心情,事情,几率,难过	happy, mood, things, odds, sad
17	42	Initiative	面对,压力,生活,健康,人生	face, pressure, life, health, life
18	11	*A Little Red Flower* (a popular Chinese movie released in 2020)	小花,一朵,千惠,小红花,病魔	little flower, a, Chie, little red flower
19	45	Suspicion of breast cancer	怀疑,焦虑症,返祖,胸痛,检查	suspicion, anxiety, revert, chest pain, examination
20	48	Other cancers	肺癌,肝癌,胃癌,肠癌,吸烟	lung cancer, liver cancer, stomach cancer, bowel cancer
21	64	Anxiety	焦虑,担心,烦躁,考研,心情	anxiety, worry, irritable, exam, mood
22	17	Metastasis of cancer cells	转移,癌症,癌细胞,患者,闫宏微	transfer, cancer, cancer cells, patient, Yan Hongwei
23	22	Weibo follows	关注,微博,抗癌,荔枝,记录	concern, microblogging, anti-cancer, lychee
24	23	Weibo usage	微博,媽媽,努力做到,更新,不想	microblogging, mom, trying to do, update, don't want
25	85	Treatment side effects	头发,假发,化疗,光头,掉头发	hair, wig, chemotherapy, bald, lose hair
26	27	Check-up	姐夫,电话,昨天,医生,回去	brother-in-law, phone, yesterday, doctor, go back
27	63	Female physiology	没事,预防,增生,例假,一去	Nothing, prevention, hyperplasia, period, a go
28	9	Public figures	陈晓旭,李明,伤官,林黛玉,李婷	Chen Xiaoxu, Li Ming, hurt official, Lin Daiyu, Li Ting
29	58	Treatment stages	化疗,第二次,第三次,结束,白细胞	chemotherapy, second, third, end, white blood cells

#### Notable Topics

##### Negative Emotions and Anger

The sentiment analysis suggested that the “no_patient” class had consistently higher negative emotions and anger than all other poster-patient relationship classes. Next, we examined the top 30 topics to identify topics with a similar pattern, which were topics 0, 2, 3, 18, 13, 23, 42, 45, 48, 64, and 113. These spanned a number of overlapping themes. Topic 0, for example, contained terms that directly expressed anger and also appeared to carry the speculation that anger is a cause of breast cancer. Similarly, topics 42, 64, and 113 comprised emotive posts about being positive or hopeful in the face of breast cancer, as well as the anxiety and stress it causes. Posts on topics 3, 48, and 63 contained physiological and medical terms, particularly cancer-related terms, their comorbidities, and their antecedents, and posts on topic 45 appeared to express anxiety at the poster facing a possible cancer diagnosis. Finally, topics 2 and 18 contained posts about the user having a nightmare about breast cancer while sleeping, and topics 13 and 20 were about cancer in everyday life. A guiding theme for these topics is that they seem to relate to the posters’ fears and anger toward cancer in general.

##### Sadness and Anxiety

Topics 26 and 58 resembled the patterns of relationship classes for sadness and anxiety, in that with the exception of the “post_user” class, the “family_members” class was more prevalent than the other poster-patient relationship classes. These topics shared a common theme, in that they discussed treatment options for breast cancer (eg, chemotherapy, immunotherapy). One explanation could be that immediate family members, as caregivers, were more concerned about breast cancer treatment.

#### Error Analysis for Machine Learning Classification

Although our classifiers predicted posts well to some extent, we noticed that some cases were mistakenly classified into other categories, according to the metrics from Tables 3 and 6. To explore the possible reasons behind this misclassification, we implemented error analysis.

We found that 1 common reason for these errors was when the patient in a post was unclear and what they said needed to be inferred through semantic understanding. In Table S3 in Multimedia Appendix 4, for example, in post I, the breast cancer patient in the post was the post author (we inferred that the patient should be the poster from reading the post), so according to our classification definition, the true label would be “post_user,” but the predicted label from our classifiers was “acquaintances.” We think that this could be attributed to a mention of a colleague at the beginning of the post and was mistakenly classified into the “acquaintances” class instead. We observed another reason for errors was when the patient was clearly mentioned but there were multiple other actors mentioned in the post as well. Such appearances can greatly affect the classifiers’ prediction. In post II, based on our understanding, the patient appeared to be the poster, but there were many other family members present (eg, father, baby, son, daughter-in-law, granddaughter, grandma). Therefore, post II was mistakenly classified into the “family_members” class instead of the “post_user” class.

## Discussion

### Principal Findings

#### Step 1: Poster-Patient Relationship Classification

We fine-tuned the pretrained language model Chinese RoBERTa on our annotations on poster-patient relationships to construct a classification model capable of identifying patients’ relationships with the posters of Weibo posts concerning breast cancer. We subsequently used those classifiers to implement a 2-stage classification process. Both classifiers performed well, and we were generally able to classify poster-patient relationships with moderate-to-high accuracy. This comprised step 1, the poster-patient relationship classification, which was essential to our research question of examining differing Weibo posting styles across poster-patient relationships.

#### Step 2: Principal Results for Sentiment Analysis and Topic Modeling

In step 2, we used sentiment analysis to compare emotion expressiveness across the 6 poster-patient relationship classes, followed by topic modeling to connect topic content with the emotional difference among identity categories in order to gain an overall understanding. Although this offers only an approximate attempt to interpret the findings of the sentiment analysis, it nevertheless offers an early window into how Weibo posts on breast cancer differ according to the relationship the patient has with the poster. Here, we remind readers that (1) sentiment analysis was calculated based on broad trends in emotion categories, in that for a specific emotion category, having a higher performance in a relationship class meant that it had a higher frequency across all data, and (2) the distribution of topics per class was performed using the corresponding frequency number of each category across all data, which effectively presented the participation for each relationship class in each topic. In other words, among the 6 relationship classes, the correspondence between each relationship class for each emotion category and the correspondence between each relationship class for each topic can only approximately connect both results to contextualize the emotion from the topic when the relevance is consistent. It does not, however, directly represent the actual relationships between topics and emotion terms, so we caution readers against overinterpreting these results.

### Anger and Negative Emotions in “no_patient” Posts

One strong result observed from the sentiment analysis was that “no_patient” posts were consistently higher on anger and negative emotions in general. Considering the topics that are more closely associated with the “no_patient” posts, our interpretation is that posts that omit explicit mentions of patients could indicate the poster’s apprehension, anxiety, or anger toward breast cancer. For example, this could come in the form of a rant. Ranting on social media is a common behavior for expressing stress and dissatisfaction with certain aspects of life. For some users, ranting on a social media platform encourages social support from other users [38] and is therefore more preferable than ranting in closed media (eg, a diary). Second, ranting on social media has a cathartic effect on the individual with regard to anger reduction [39]. This may thus be a constructive outlet [40] for posters to reduce their negative emotions when feeling particularly angry or anxious toward breast cancer. In these types of posts, we think that the poster may omit explicit mentions of the patient, as these posts are not necessarily of an autobiographical nature but of an expressive nature instead (eg, flow-of-thought writing) and may occur in any situation in which the poster may have a reason to be angry at cancer. For example, posters may be angry at a diagnosis (or prospect) of cancer in themselves or their loved ones, or they may be angry at the problems in society that arise from cancer and associated treatments, which do not necessarily need a target patient.

### Sadness and Anxiety in “family_members” Posts

In contrast, sadness and anxiety were consistently higher in posts where close family members (eg, parents) were the patients. This also corresponded with more mentions of treatment options. Past research has documented the significant emotional burden placed on close family members as caregivers of patients with cancer [41]. Moreover, this could be exacerbated by cultural factors: family members are more closely linked to the concept of the self in China, which is largely consistent with interdependent self-construal and collectivistic cultural orientation [42]. In Chinese society, the burden of caregiving often falls to family members, such as adult children [43]. Moreover, (lack of) familial support has been linked to depression and loneliness in elderly Chinese, suggesting the importance of family ties as relational aspects of one’s well-being (eg, interdependent happiness [44]). This may explain the greater mentions of treatment options, and the sadness and anxiety, in Weibo posts where the patient was identified as a family member of the poster; the patient was considered relationally closer and more important to their self-identity, and the poster would also more likely be engaged in caregiving.

This could also be a unique cultural aspect of Chinese individuals. Previous studies have shown that American individuals (elderly) have more independent self-construal, and familial ties, being obligatory, are often less important to the self than friendship ties [44,45]. However, more research is needed to examine similar posts on Western social media platforms for proper cross-cultural examination.

### Implications and Future Directions

Our research identified how emotion expression and content change according to the poster’s relationship with the patient, and aligns closely with past research on the stresses and risks family caregivers face for depression and anxiety disorders [36]. This is particularly exacerbated in Chinese culture, where the strain of caregiving is often intensified through cultural norms surrounding filial piety [46]: this means that caregivers often must maintain a patient and positive outlook when interacting with their patients so as not to put an additional burden on the patients. Moreover, discussions about cancer are often seen as taboo in Chinese society, so caregivers cannot easily access social support from their friends and family. However, as social media provides an opportunity for sharing experiences and outreach, it holds immense potential for community building and social support, particularly for familial caregivers (see Ref. [47]). Therefore, we think that social media opens up new opportunities for caregivers (and patients) to seek social support, with reduced fears of breaking social norms and facing judgment from their community. This may even be above and beyond the benefits of social media–based social support in comparatively open Western societies, and we encourage further studies to examine how Chinese internet spaces should be designed to facilitate such social support.

### Limitations

To obtain our target data set (long narratives pertaining to breast cancer), we needed to contextualize our initial Weibo queries with additional keywords, in this case “sadness.” Although this enhanced the quality of our data set, it would have biased the data toward more negative sentiments. Nevertheless, despite the overt bias toward negative posts in our sample, significant differences were still observed in poster-patient relationship classes.

During our classification process, we constructed 2 classifiers based on language models. For the binary classifier, the model reached an *F*_1_-score of 0.9, and for the multiclass classifier, the model reached an *F*_1_-score of 0.8 on average. Although these values are good, there is still some room for improvement for our classifiers. One possibility would be to use a better model for multiclass classification.

In sentiment analysis, we implemented a LIWC-based tool based on the lexical matching of terms for word frequency. Moreover, since only 5 broad affective categories (positive emotions, negative emotions, anger, anxiety, and sadness) were included in this tool, we focused only on these in our study. We think that with newer and more powerful sentiment analysis tools and a larger number of affect categories, the accuracy and granularity of sentiment analysis can be further improved for more valuable insight from the text corpus.

For topic modeling, we used the BERTopic tool to cluster topics, and we found that all the generated topics only had subtle distinctions, which led to several overlaps in similar content among topics. For a better understanding of topics, a qualitative assessment of posts would have yielded deeper insights into the data, but this would not have been practical, given the size of the data set.

### Conclusion

In this paper, we studied breast cancer–related narratives on the Chinese social media platform Weibo. Using a pretrained transformer language model (Chinese RoBERTa) as the base model, we fine-tuned 2 models on an annotated subset of the data to classify poster-patient relationships in those posts in a sequential process. Ultimately, we classified all posts according to the identified poster-patient relationships (post_user, family_members, friends_relatives, acquaintances, heard_relation, or, if no patient was identified, no_patient).

Next, we implemented sentiment analysis. We used the Chinese LIWC lexicon to examine the sentiment among 6 categories, focusing on positive emotions, negative emotions, anger, anxiety, and sadness. Through statistical comparisons, we found that emotional expressions present differences among different poster-patient relationship classes. For example, the “no_patient” class had a significantly higher level of anger compared to other classes.

To contextualize these results, we also conducted topic modeling using BERTopic. This showed that posts had different topical content according to the different poster-patient relationships. For example, the “no_patient” class presented more anger in the discussions, while the “family_members” class showed more care for hospitalization and treatment. In sum, our results indicate that patient-poster relationships show differing content and language on Weibo.

## Appendix

Multimedia Appendix 1 Annotation sample.

Multimedia Appendix 2 Examples of automatic summarization of long Weibo posts.

Multimedia Appendix 3 Top 30 terms of top 30 topics from topic modeling.

Multimedia Appendix 4 Examples of error analysis.

## Abbreviations

BERT: Bidirectional Encoder Representations from Transformers
DSCF: Dwass-Steel-Critchlow-Fligner
LIWC: Linguistic Inquiry and Word Count
NLP: natural language processing
RoBERTa: A Robustly Optimized BERT Pretraining Approach
WHO: World Health Organization

## Data Availability

As data contain social media posts that may be linked to individuals, data will be made available upon request to the authors.
