# Supplementary material for: Differing Content and Language Based on Poster-Patient Relationships on the Chinese Social Media Platform Weibo: Text Classification, Sentiment Analysis, and Topic Modeling of Posts on Breast Cancer
Source: JMIR Cancer. 2024 May 9;10:e51332. doi: 10.2196/51332 (PMC11117131; doi:10.2196/51332)
Supplement: Multimedia Appendix 1 [file cancer_v10i1e51332_app1.docx]

**Table S1.** Annotation sample.

| Weibo posts (Original Chinese) | Weibo posts (Machine Translation to English) | Label |
| --- | --- | --- |
| 去让自己相信自己得了癌是一件漫长的事情，即使有两份确诊报告摆在我的面前,当第二份报告我拿在手里，我心里默念三遍阿弥陀佛，打开看到结果和第一次结果一样的时候，我心里咯噔一下，我被确诊了乳腺癌！我能做的就是告诉自己，接受它，面对它，然后打败它！ | It was a long time to convince myself that I had cancer, even though I had two reports in front of me. When I held the second report in my hand, I chanted Amitabha three times in my heart, and when I opened it and saw that the result was the same as the first result, my heart thumped, I was diagnosed with breast cancer! All I could do was tell myself to accept it, face it, and beat it! | post_user |
| 妈妈确诊乳腺癌的第三天，除了哭什么都做不了，不能替她生病，不能替她分担痛苦，确诊之前一家人高高兴兴的，我从来不奢求我的妈妈有多么的有钱，只想她平平安安健健康康的，可为什么那么难呢，妈妈一生多灾多难，所有的痛苦都承受了，我不怪老天不公，只怪老天为什么不放过她，如果可以，我希望生病的是我自己，如果能让妈妈健康，要我做什么都可以，只希望老天能善待我。 | On the third day of my mom's breast cancer diagnosis, I couldn't do anything but cry, I could not share her sickness and pain, my family was happy before the diagnosis, I never wanted my mom to be rich, I just wanted her to be safe and healthy, but why was it so hard? I don't blame God for being unjust, I just blame God for not letting her go. If I could, I wish I was the one who was sick, if I could make my mom healthy, I could do anything, I just hope God would treat me well. | family_members |
| 难得有一会的空闲时间，想想人的一生做点自己喜欢的事真的挺难。昨天惊闻一个好朋友的了乳腺癌小女儿才两岁。好可怕！没有妈妈的孩子，想想就心疼。希望所有的孩子都有妈妈爱，妈妈们身体健康。希望人类早日攻克癌症！祝福我的好朋友早日康复！ | It is rare to have a moment of free time to think about how hard it is to do something you love in your life. Yesterday, I was shocked to hear that a good friend had breast cancer and her little daughter was only two years old. How terrible! It hurts to think of a child without a mother. I hope all children have their mothers' love and mothers are in good health. I hope mankind will overcome cancer soon! Wish my good friend a speedy recovery! | friends_relatives |
| 今天突然得知以前的一个同事癌症到了最后的时期。三年前检查出来了乳腺癌，切的时候发现扩散到了淋巴一部分就一并切了，去年发现扩散到了骨头，这两天说是已经不认识人了。他的女儿才五年级，还有年迈的父母，不过还好她不是独生子女。有时候我在想这样和离婚失去父母一方，哪个对孩子的影响小一些？ | Today I suddenly learned that a former colleague has reached the final stage of cancer. Three years ago, she was examined for breast cancer, and when she was cut, she found that it had spread to the lymphatic part and was cut together. Last year, she found that it had spread to the bones, and in the past two days, she said that she did not recognize people. His daughter is only in the fifth grade and has elderly parents, but fortunately she is not an only child. Sometimes I wonder which has less impact on the child, this or losing one parent to divorce? | acquaintances |
| 和同事闲聊，得知她同学经常生前男友的气，年纪轻轻就得了乳腺癌早期。感叹现在的癌症越来越年轻化以后更要好好爱自己的同时，暗自庆幸2年前的决定，一个男的时常让你生气，连你的心情都不顾及，还指望他以后善待你？一段关系，开心舒适最重要！这年头，还是保命最要紧！顺带嘲讽一下牛哥最近可爱肉见涨，自带喜感。 | I was chatting with a colleague and learned that her classmate was often angry with her ex-boyfriend and got early stage breast cancer at a young age. She sighed that cancer is getting younger and younger, and that she should love herself more, but she was glad that she made the decision 2 years ago. A relationship, happy and comfortable is the most important! These days, it's important to save your life! Incidentally, mock the cattle recently cute meat to see the rise, bring their own sense of comedy. | heard_relation |
| 结婚生小孩对我而言，真的是让我瞬间老了十岁的选择。每每一次郁结，我都觉得乳腺癌离我又近了一步。那些婆婆妈妈琐琐碎碎的生活，硬生生把我变成一个每天只会抱怨和唠唠叨叨黄脸婆。呵呵。只要不生活在同一个屋檐下面对屎尿屁的生活，谁在外面还他妈不是个人见人爱的小仙女呢。 | Getting married and having children was really a choice that instantly aged me by ten years for me. Every time I got depressed, I felt that breast cancer was one step closer to me. The mother-in-law's trivial life has turned me into a yellow-faced woman who only complains and nags every day. Oh. As long as you don't live under the same roof to face the shit life, who is not a f****** fairy outside. | no_patient |
