# Supplementary material for: Differing Content and Language Based on Poster-Patient Relationships on the Chinese Social Media Platform Weibo: Text Classification, Sentiment Analysis, and Topic Modeling of Posts on Breast Cancer
Source: JMIR Cancer. 2024 May 9;10:e51332. doi: 10.2196/51332 (PMC11117131; doi:10.2196/51332)
Supplement: Multimedia Appendix 2 [file cancer_v10i1e51332_app2.docx]

Table I: some samples of the automatic summarization

| Sample | Original post | Summarized post |
| --- | --- | --- |
| 1 | 以为这么多年了，在这个生老病死已经变得太稀松平常的科室，我已经丧失共情能力了。但是早上看到一个从我刚开始工作到现在和我关系一直特别特别好的爷爷的孙子发的朋友圈，得知爷爷今天走了，心里真的揪着疼。爷爷是个离休干部，基本一年有6个月呆在医院，从我刚工作那会，他好像就已经在病房了。他住院，奶奶就陪着一起住，老两口对我超好，有啥好吃的都偷偷塞给我，还给我取了个昵称“小猴子“。这次住进来其实情况一直不太好，刚好这个月我是他的主管护士，所以是眼见着他慢慢变差。我记得进icu那天白天，我一个早上问了他无数次还记不记得我是谁，一方面是出于工作需求需要确定他还有没有意识，一方面是真的怕他不记得我了。他从刚开始还会回答我“你是小猴子呀”，到后来吞吞吐吐的“小.猴.子”，再到后来只会吃力的点点头，一直到最后已经不懂我在说什么，也没力气再回应我了。我感觉自己的心都在一点点往下坠。因为见过太多这种情况了，我太知道接下来会发生什么。那天下午也是我亲自把爷爷送进icu，送进去的时候，我偷偷在爷爷耳朵边说了句“爷爷别怕，过两天我们就回来”。后来自己回来科室的路上没忍住哭了。我一直是个对工作很认真的人，不管是工作本身，还是和病人的相处，我都尽我所能。我付出真心，大部分病人也会给我同样的反馈，依赖我信任我，导致我对他们会产生特殊的感情。很怕他们情况变的不好，甚至在我面前离开。每离开一个，我都会难受很久。有时候站在他们之前住过的病床，我都会有那么一瞬间的恍惚，感觉他们都还在。我还老是念叨着有些老病人很久没来住院了，不知道该开心还是难过，开心他们可能一切都好，不需要再来医院，难过也有可能他们已经不在人世。那天看人间世，大学老师得了乳腺癌那一期，刚开始我以为自己不会太触动，但是后来看着看着还是哭的泪流满面。因为它太常见了，常见到我在面对那些来科室化疗靶向的病人，我的内心都快没有多少波动了，但我忘了，乳腺癌也是癌症，每一个乳腺癌患者的背后其实都像这个大学老师一样，我看不到她们除了按时来医院检查抽血化疗之外还做了多少努力。我有点自责，感觉自己做的不够，有时候态度是不是不太好。不知道自己到底在说什么，这个点了脑子里全是和这个爷爷还有奶奶相处的每一幕。记得他们叫的每一声小猴子，记得他们好多次把好吃的塞进我的口袋并且嘱咐我别让其他同事看见，记得他们千方百计的骗我去病房和他们孙子见面，非要把孙子介绍给我，记得他们出院之后还特意给我打电话问问我的近况。有时候真的希望自己是个没有感情的杀手，这样就不会老是这么容易难。  I thought I had lost the ability to empathize with people in this department where death and old age have become too commonplace after so many years. However, when I saw a friend's circle sent by the grandson of my grandfather, who has been very close to me since I started working, and learned that my grandfather had left today, my heart really ached. Grandpa is a retired cadre, basically 6 months a year in the hospital, from the time I first worked, he seems to have been in the ward. He was hospitalized, Grandma stayed with him, the old couple was super nice to me, what's good to eat are secretly stuffed to me, but also gave me a nickname "little monkey". This time, he has not been doing well, and this month I was his nurse in charge, so I saw him slowly deteriorate. I remember during the daytime on the day he entered the ICU, I asked him countless times in the morning if he still remembered who I was, partly out of work needs to make sure he was still conscious, and partly because I was really afraid that he wouldn't remember me. At first he would answer me, "You're a little monkey", but later he stammered, "Little... monkey...". Monkey. Zi", and then only nodded his head with great effort, until finally he didn't understand what I was saying and didn't have the strength to respond to me anymore. I felt my heart falling a little bit. Because I had seen this kind of situation too many times, I knew too well what would happen next. I was the one who took my grandfather to the ICU that afternoon, and I secretly whispered in his ear, "Don't be afraid, Grandpa, we'll be back in a couple of days. Later, I couldn't hold back my tears on the way back to the department. I've always been a very serious person about my work. Whether it's the work itself or my relationship with patients, I always do my best. I give my heart, and most of my patients give me the same feedback, relying on me and trusting me, causing me to develop special feelings for them. It's scary to think that they will get worse or even leave in front of me. Every time I leave one, it's hard for me for a long time. Sometimes when I stand in the hospital beds where they have stayed before, I have a moment of trance and feel that they are all still there. I also keep reading that some old patients haven't come to be hospitalized for a long time, and I don't know whether to be happy or sad. I am happy that they may be fine and don't need to come to the hospital anymore, and I am sad that they may not be alive anymore. The other day, I was reading about the world on earth, and my college teacher got breast cancer, and at first I thought I wouldn't be too touched, but then I cried and cried and cried. Because it is too common, common to me in the face of those who come to the department of chemotherapy targeting patients, my heart is not much fluctuation, but I forgot, breast cancer is also cancer, every breast cancer patients behind in fact, like this college teacher, I can not see them in addition to come to the hospital on time for checkups, blood draws, chemotherapy, in addition to how much effort they have done. I'm kind of beating myself up, feeling like I'm not doing enough, and if I don't have a good attitude sometimes. I don't know what the hell I'm talking about, but at this point my mind is filled with every scene with this grandpa and grandma. I remember every time they called me Monkey, I remember how many times they put good food into my pocket and told me not to let other colleagues see, I remember how they tried to trick me into going to the hospital room to meet with their grandchildren and introduced them to me, I remember how they called me after they were discharged from the hospital to ask me how I was doing. Sometimes I really wish I was an emotionless killer so it wouldn't always be so easy and difficult. | 有时候态度是不是不太好,刚开始我以为自己不会太触动,这次住进来其实情况一直不太好,我一个早上问了他无数次还记不记得我是谁,这样就不会老是这么容易难,我还老是念叨着有些老病人很久没来住院了,一方面是真的怕他不记得我了,不需要再来医院,我都会难受很久,他从刚开始还会回答我“你是小猴子呀”,感觉他们都还在,每一个乳腺癌患者的背后其实都像这个大学老师一样,开心他们可能一切都好,但是早上看到一个从我刚开始工作到现在和我关系一直特别特别好的爷爷的孙子发的朋友圈,那天下午也是我亲自把爷爷送进icu,我记得进icu那天白天,后来自己回来科室的路上没忍住哭了,不知道该开心还是难过,记得他们好多次把好吃的塞进我的口袋并且嘱咐我别让其他同事看见,我太知道接下来会发生什么,我都会有那么一瞬间的恍惚,一方面是出于工作需求需要确定他还有没有意识,记得他们叫的每一声小猴子,感觉自己做的不够,一直到最后已经不懂我在说什么,不知道自己到底在说什么,记得他们千方百计的骗我去病房和他们孙子见面,我偷偷在爷爷耳朵边说了句“爷爷别怕,这个点了脑子里全是和这个爷爷还有奶奶相处的每一幕,难过也有可能他们已经不在人世,大部分病人也会给我同样的反馈。  Sometimes the attitude is not too good, at first I thought I would not be too touched, this stay in fact, the situation has not been very good, I asked him countless times in the morning still remember who I am, so that it will not always be so easy to be difficult, I also always read some of the old patients have not come to the hospital for a long time, on the one hand, I am really afraid that he does not remember me, do not need to come back to the hospital, I will be difficult for a long time, he from the beginning He would still answer me "You are a little monkey", and I felt that they were all still there, and that every breast cancer patient was like this university teacher, happy that they might be all right. However, in the morning, I saw a friend's circle posted by a grandpa's grandson, who had been on very good terms with me since I first started working, and I personally brought my grandpa to the ICU that afternoon. I personally put my grandfather into the ICU, I remember the day I went into the ICU, then I couldn't hold back my tears on the way back to the department, I don't know if I should be happy or sad, I remember how many times they stuffed goodies into my pockets and instructed me not to let other colleagues see, I knew too well what was going to happen next, I would be in a trance for a moment, partly out of the demands of the job to make sure that he was still conscious, partly out of the demands of the job to make sure that he was still conscious, partly out of the demands of the job to make sure that he was still conscious, partly out of the demand for the work to be done, and partly out of the need for the work to be done. I remember every time they called the little monkey, I feel that I have not done enough, until the end of the day, I do not understand what I am saying, do not know what I am talking about, I remember that they tried to trick me to go to the ward and their grandson to meet, I secretly in the ear of the grandpa said "Grandpa do not be afraid of this point of time in the head is full of grandpa and grandma with every scene, sadness has been Most of the patients would give me the same feedback. |
| 2 | 小感慨一下！今天两台乳腺手术，年龄不相上下的两位阿姨！都有不同的经历！第一个阿姨在等病理报告时说，两年前30岁的独生女儿被查出乳腺癌，她说当时她都崩溃了！就是现在她也不敢陪女儿去复查，怕听到结果！今天自己来做手术，全家都来了，她说她不愿意让女儿来！女儿在她进手术室时给她打电话，女儿没敢和她直接说，是外孙女和她说姥姥不要害怕，你要加油啊！我和妈妈在家等你！我听完心里不是滋味！好在结果不错！第二个阿姨我前一天访视时就看见老两口在病房，我问有其他家属吗？两个老人说孩子们都忙没让他们知道，我这是小手术不要紧！今天早上进手术室后，阿姨说我很紧张，我怕我的不好！昨天医生交代的我有点害怕了！我说没事阿姨，既来之则安之！没事，能做手术的都不是事！阿姨说谢谢你啊！也是再等病理报告时（术中冰冻需要大约30-40分钟）阿姨告诉我们，她家两个孩子，一丫一小。她很骄傲，两个孩子都不用她操心！丫头在青岛，小两口开个面包店生意很好可忙了，孩子还小就没让她知道，怕知道了不管不顾的跑回来！小子在哈工大读博！也没告诉他，怕影响他学业！所以老两口就自己来了！病理结果不太好！我给阿姨扎针时，阿姨问为什么扎针，我说得重新取块病理，因为之前取病理时局麻药扎的有点多，您现在就得全麻了！我扎完针，来到头侧安慰她时，阿姨说我都明白！你不说我也懂！在给大爷看病理时，大爷说没想到是这个结果！就以为是个小手术呢！手术结束后，我送阿姨回病房时，大爷在阿姨旁边说没事了！好了！都切了就放心了！我看着有点心酸，我说大爷您不行告诉家里人吧，您一个人照顾不了阿姨！大爷说，没事，我能照顾她，不给孩子添麻烦！瞬间就觉得父母的伟大！我帮着整理床，帮着抬，能帮的我尽量都帮着弄舒适了！祝福今天这两位母亲，健康快乐！⬇️⬇️⬇️下面的图就能看出母亲的不容易！母亲真的是个伟大的职业！也祝福所有的母亲健康快。  A small sentiment! Two breast surgeries today, two aunts of similar ages! Both had different experiences! The first aunty, while waiting for the pathology report, said that her 30-year-old only daughter was diagnosed with breast cancer two years ago, and she said she was devastated at the time! It's now that she doesn't dare to accompany her daughter to the review for fear of hearing the results! Today she came in for surgery by herself, with the whole family present, and she said she didn't want her daughter to come! Her daughter called her when she was in the operating room. She didn't dare to talk to her directly, but it was her granddaughter who said, "Don't be afraid, grandma, don't be afraid, you have to work hard! Mom and I are waiting for you at home! I was not happy to hear that! The good thing is that it turned out well! When I visited the second aunt the day before, I saw the elderly couple in the ward and asked if there were any other family members. I asked if there were any other family members. The two old people said that their children were busy and didn't let them know that I was having a minor surgery and that it didn't matter! This morning after entering the operating room, auntie said I was very nervous, I'm afraid I'm not good! I was a little scared of the doctor's explanation yesterday! I said it's okay, Auntie, I'm fine with it! It's okay, it's not a problem to be able to do surgery! Auntie said thank you! While waiting for the pathology report (it takes about 30-40 minutes for the intra-operative freezing), Auntie told us that her family has two children, one girl and one boy. She is very proud that both kids don't need her to worry! The girl in Qingdao, the young couple opened a bakery business is very good can be busy, the child is still small, did not let her know, for fear of knowing no matter what to run back! The boy in the Harvard University to read the doctorate! I didn't tell him, for fear of affecting his studies! So the old couple came by themselves! The pathology results are not good! When I gave the aunt a needle, she asked why I did it, and I told her that I had to take a new piece of pathology because I had used a little too much local anesthetic when I took the pathology, so now you'll have to go under general anesthesia! When I finished the needle and came to the side of the head to comfort her, Auntie said I understand everything! You don't have to say I understand! When showing moncler outlet store the pathology, moncler outlet store said I didn't expect this result! Just thought it was a minor surgery! After the surgery, when I sent Auntie back to the ward, moncler outlet store next to Auntie said it was okay! It's okay! It's a relief to have it all cut! I was a bit saddened by this. I said, "You can't tell your family, you can't take care of your aunt alone! I can take care of her, and I don't want to cause any trouble for the kids," he said! I felt the greatness of the parents! I helped to make the bed, helped to lift it, and tried to make it as comfortable as I could! Blessings of health and happiness to these two moms today! ⬇️⬇️⬇️ The picture below shows how difficult it is for mothers! Motherhood is really a great profession! I wish all mothers good health and happiness. | 我说没事阿姨,两个老人说孩子们都忙没让他们知道,大爷在阿姨旁边说没事了,第一个阿姨在等病理报告时说,阿姨说我都明白,她说她不愿意让女儿来,两个孩子都不用她操心,能做手术的都不是事,阿姨说我很紧张,病理结果不太好,女儿没敢和她直接说,在给大爷看病理时,阿姨说谢谢你啊,你不说我也懂,大爷说没想到是这个结果,您一个人照顾不了阿姨,她说当时她都崩溃了,我给阿姨扎针时,就以为是个小手术呢,大爷说,我这是小手术不要紧,祝福今天这两位母亲,阿姨问为什么扎针,因为之前取病理时局麻药扎的有点多,第二个阿姨我前一天访视时就看见老两口在病房,我说大爷您不行告诉家里人吧,年龄不相上下的两位阿姨,不给孩子添麻烦,能帮的我尽量都帮着弄舒适了,也是再等病理报告时（术中冰冻需要大约30-40分钟）阿姨告诉我们,怕听到结果,⬇️⬇️⬇️下面的图就能看出母亲的不容易,我送阿姨回病房时,今天两台乳腺手术,怕影响他学业,女儿在她进手术室时给她打电话,就是现在她也不敢陪女儿去复查,怕知道了不管不顾的跑回来,也祝福所有的母亲健康快,她家两个孩子,今天自己来做手术,孩子还小就没让她知道,手术结束后,是外孙女和她说姥姥不要害怕。  I said it's fine,moncler outlet, the two old people said the children are busy did not let them know,moncler outlet, next to the aunt said it's fine,moncler outlet online, the first aunt waiting for the pathology report,moncler outlet online, the aunt said I understand,moncler outlet online, she said that she was not willing to let her daughter, the two children do not need to worry about her,moncler outlet store, the surgery is not a matter of course,moncler outlet online, I was very nervous,moncler outlet online, the results of the pathology is not very good, her daughter did not dare to talk to her directly, in the moncler outlet sale pathology,moncler outlet online, the aunt said thank you,moncler outlet online. The first thing you need to do is to get a good deal of money from the company. moncler outlet online The first thing you need to do is to get a good deal of money from the company. moncler outlet online The first thing you need to do is to get a good deal of money from the company. moncler outlet online The first thing you need to do is to get a good deal of money from the company. moncler outlet online The first thing you need to do is to get a good deal of money from the company. moncler outlet store The first thing you need to do is to get a good deal of money from the company. moncler outlet store The first thing you need to do is to get a good deal of money from the company. moncler outlet store The first thing you need to do is to get a good deal of money from the company. moncler outlet online Ward, I said moncler you can not tell the family, the age of the two aunts, do not give the child trouble, can help me try to help get comfortable, but also to wait for the pathology report (intraoperative freezing takes about 30-40 minutes) aunts told us, afraid to hear the results,⬇️⬇️⬇️ the following picture can be seen the mother of the mother's uncomplicated, I sent aunts back to the ward, the two breast surgeries today for fear of affect his studies, her daughter in her into the operating room when she called her, is now she did not dare to accompany her daughter to review, for fear of knowing no matter what run back, but also blessed all the mothers health fast, her family two children, today they came to do the surgery, the child is still small, did not let her know, the end of the operation, is the granddaughter and she said grandma do not be afraid, the operation is over. |
| 3 | 今天小姨给我们讲了她好朋友的事。事情围绕着一对从小玩到大的闺密，下边称A和B。八九岁刚读书的时候，A和B相识相知，然后成为好朋友。A和B都是普通家庭，父母双全。有点特别的是，A的姐姐是个苦命又要强的女人，年轻时她姐姐爱上了一个男人，二十来岁嫁给了他，但结婚不到两年，才生下女儿没多久，就发现丈夫出轨，并且小三已经挺着大肚子。A的姐姐当机立断，离婚，再也不嫁，把自己当成男人使，二十来岁最美好的年纪，帮着父母打理一家小店铺，自己养女儿，帮着养父母（父母的身体都不好），还有养两个妹妹（其中一个妹妹就是A）。这家小店铺，在本地的大医院正门附近，卖一些病人住院需要的生活用品，生意慢慢经营着，倒也支撑了家里两个妹妹读了书。在往后十几年期间，A和B关系越来越亲密，彼此之间毫不保留，毫无秘密，也经常来往对方的家，吃喝玩乐全在一起。两家人都认识，都像多了一个女儿一般。时光荏苒，A和B都嫁了人，嫁得都还算不错，A的老公家那边稍微有点小钱，B的老公家那边稍微有点小权。那会A的父母也已经年迈，并且病痛缠身，A的姐姐帮着打理生意十多年，慢慢也有了起色——她和医院的一些医护人员混熟了，医院内部开始直接和她联系采购日用品，靠着这条关系有时候一个月可以赚到一万来块（在18线小城市，一个女的做小生意能月赚一万，对于普通家庭算是很满足的数目了），但是呢，因为父母身体原因，加上养女儿和维持家常，每个月所剩也无几。日子就这样继续过着。时间就到了两年前，A的姐姐查出来得了乳腺癌，同时，医院的院长选了别人当采购，于是A家姐姐失去了这份珍贵的工作，不幸中的万幸是，癌症是良性的，做完手术也就慢慢缓了过来，那会上有父母，下有女儿，手术又花了不少钱，工资更是降到月入不到两千。生活真是苦到让人心酸的程度。而A嫁的还可以，所以也会补贴娘家，那会B的老公说是在做点小生意，资金紧张的时候，B也会向A借钱，A本身不是大方的人，从来不花不该花的钱，但是对于家里和B的请求，一直会毫不犹豫地答应，一万两万，借来借去让B家慢慢周转。后来，B的母亲病逝，A甚至都去B家帮着对方母亲穿寿衣，帮洗漱，再后来A的母亲也病逝，也是互帮互助…闺密啊，做到这个份上，毫无保留，相知相惜，也的确是让人羡慕了。然后就到了今年，A家的老父亲身体更加不好，隐隐有灯尽油枯之势，A和A的姐姐少不了又是一顿照顾忙活。而B家的生意如日中天，不过却在最近遇到了个小麻烦——在采购上一直得不到客户的满意，于是B就去问A，你家以前也是采购的，我们这边也和医院有采购的合作，现在想买一款医用袋子一直买不好，你们以前在哪里买，买多少钱，批发又多少钱，卖给医院补贴多少钱？之前有说过，她们从小彼此就毫无秘密，B是知道A家打理生意的所有事的，也知道A家从无到有，又从有到无，后来B的老公也开始做生意做采购，其实也是受A家的影响。A听完B的困扰，也就倾囊而出，把相关的事都讲的明明白白。B照着A的意见去做，诶，客户那边就满意了。A的老父亲病得很重，A和姐姐一起去照顾，A想起这事，也就顺口聊了一会，A的姐姐听完，突然就觉得有些奇怪，就问了下有没有B家老公的照片，A就发给她姐姐，她姐姐毕竟也曾在医院做了十几年生意，和医护人员的关系还是有的，就拿B家老公的照片去问医护人员：你们这边最近是不是有这个人在帮忙采购？医护人员一看，哎呀，我们采购是这个人，不过不是最近的，两年前就是他呢！他就是我们院长调来代替你的人呀！……——————————我个人真的难以想象她姐姐当时的心情，更难以想象A知道真相后的心情。——————————两年前在A家最苦最难的时候——父母病重，姐姐癌症，也是B完完全全知道这些事的时候，她竟凭着早期对A家生意的了解，又借着老公家的一点小权，协助自己的老公亲手抢了A家的生意，并且一直隐瞒到底。期间赚钱时不吱声，拮据时又向A借钱周转……A知道真相后几近崩溃，这真的是活生生的——把自己卖了还帮着别人数钱。而B，像是吃着人血馒头，却毫无愧疚。事情捅破时，B还欠着A一万多，A的姐姐气愤归气愤，倒也还算看得开，A就彻底沦陷在“被最亲的闺密背叛”和“害苦了自己的姐姐”的自责里走不出来，整日以泪洗面，吃不下睡不着，一下子老了十岁。而这一切，换来B带着无奈的一句：这社会谁不想赚钱啊…——————————我小姨和她们俩，加上其他几个人都是好朋友，已经有二十几年的交情，而A和B，从十岁左右到现在年近半百，已经认识了快四十年。她们有一个微信群，早上小姨收到她们群里的信息，A发了很多语音。默默地讲这件事，讲到后边一直哭，语音里尽是嘶哑和无力的抽泣：“你怎么可以这样啊……”“我们三十多年的感情，我对你可是真的比姐妹还亲啊……”“你是真的害苦了我的姐姐啊……”“你这真的是拿了一把刀，狠狠地插进我的心了啊！……”——————————这世间，可怕的不是鬼神，有时候真的是人。  Today my sister-in-law told us about her best friend. The story revolves around a pair of girlfriends who have played together since childhood, hereinafter referred to as A and B. When they were eight or nine years old and just studying, A and B met each other and became good friends.A and B are both from ordinary families with two parents. A little special, A's sister is a bitter and strong woman, young her sister fell in love with a man, twenty to marry him, but married less than two years, only to give birth to a daughter not long, found that her husband cheated on her, and the little three have been quite pregnant. A's sister made the decision on the spot, divorced, and never married again, as a man to make their own twenty years of age the best of the best age, to help their parents manage a small store, to raise their daughter, to help raise a small store, to help raise a small store, to help raise the daughter, to help raise a small store, to help raise a small store, to help raise a small store, to help raise a small store. The shop is the best place in the world to raise a daughter, to help support her parents (both of whom are in poor health), and to support her two younger sisters (one of whom is A). This small store, near the main entrance of the local hospital, sold some of the daily necessities needed by the patients hospitalized, and the business was slowly operated, but also supported the family's two younger sisters to go to school. In the next ten years, A and B relationship is getting closer and closer, each other without reservation, no secrets, but also often to and from each other's homes, eating, drinking and playing all together. Both families knew each other and were like having a daughter. Time flies, A and B are married, married are quite good, A's husband's side of the family a little bit of money, B's husband's side of the family a little bit of power. At that time A's parents have been old and sick, A's sister to help take care of business for more than 10 years, slowly also have a good - she and some of the hospital medical staff to mix well, the hospital began to contact her directly within the procurement of daily necessities, relying on this relationship sometimes a month you can earn 10,000 yuan (in the 18th line of the city, a woman doing small business can earn 10,000 yuan a month). (In a small 18-tier city, a woman doing small business can earn 10,000 dollars a month, for the average family is considered a very satisfying number), but because of the parents' health reasons, coupled with the raising of daughters and maintenance of the family routine, every month there is little left. The days went on like this. Time came to two years ago, A's sister found out that the breast cancer, at the same time, the hospital director chose someone else as a procurement, so A's sister lost this precious job, unfortunately, fortunately, the cancer is benign, after the operation also slowly slowed down, then there will be parents, under the daughter, the operation cost a lot of money, the salary is down to less than two thousand a month. Life is really bitter to the degree of heartbreaking. And A married can, so will also subsidize the family, that will B's husband said to do some small business, money is tight, B will also borrow money from A, A itself is not a generous person, never spend money should not be spent, but for the family and B's request, has been will not hesitate to agree to 10,000 to 20,000, borrowing and lending to the family to let the B slowly turnover. Later, B's mother died, A even went to B's home to help each other's mothers wear birthday clothes, help wash, and then A's mother also died, but also to help each other ....... girlfriends, ah, to do this part, without reservation, know each other, but also is indeed enviable. Then came this year, A family's old father's health is even worse, vaguely have the trend of exhaustion, A and A's sister is inevitably another meal care busy. B's business is booming, but recently encountered a small problem - in procurement has not been satisfied with the customer, so B went to ask A, your family used to be purchasing, our side and hospitals have purchasing cooperation, and now want to buy a medical bag has been to buy bad, where you used to buy, how much money to buy, wholesale! As I said before, they grew up with no secrets from each other, B is aware of all the things that the A family takes care of business, and also know that the A family from scratch, and from scratch, and then B's husband also began to do business as a purchasing, in fact, also by the influence of the A family. A listen to B's trouble, but also to pour out the relevant things are clear. B according to the A's advice to do, eh, the customer side was satisfied. Old father is very sick, A and sister together to take care of, A remembered this, but also in passing chat for a while, A's sister heard, suddenly felt a little strange, asked if there is a picture of the husband of the B's, A sent to her sister, her sister, after all, has been in the hospital for more than ten years in the business, and the relationship between the medical staff is still there, take the picture of the husband of the B's to ask the medical staff: Is this person on your side recently to help with procurement? Is this person helping with purchases? The medical staff took a look, oops, our procurement is this person, but not recently, two years ago it was him! He is the person our dean transferred to replace you! ......-------- --I personally have a really hard time imagining how her sister must have felt at the time, and even harder to imagine how A must have felt when she learned the truth. ---------- Two years ago in A's family's most bitter and difficult time - parents are seriously ill, sister's cancer, but also B complete knowledge of these things, she went so far as to rely on the early understanding of A's business, and borrowed a little bit of her husband's family's small power, to assist their own husbands personally robbed A's family's business, and has always been hidden to the end. During the period of making money when not squeaking, when in straitened circumstances and borrow money from A turnover ......A know the truth nearly collapsed, this is really alive - to sell themselves still help others count money. And B, like eating human blood buns, but no guilt. Things broke, B still owes A more than 10,000, A's sister angry to angry, but also quite open, A completely fell into the "betrayal of the closest girlfriends" and "suffer their sister" in the blame can not come out, all day long in tears, can not eat I can't eat, I can't sleep, and I've aged ten years. And all this, in exchange for B with a helpless sentence: this society who do not want to make money ah...------- --- My sister-in-law and the two of them, plus a few others are good friends, has been more than twenty years of friendship, and A and B, from about ten years old to now nearly half a hundred years, has known almost forty years. They have a WeChat group, and in the morning my sister-in-law received a message from their group, and A sent a lot of voice. Silently speak about this matter, speak to the back has been crying, voice all hoarse and powerless sobbing: "How can you do this ah ......" "Our thirty years of feelings, I'm really close to you than sisters ah ......" "You are really harming my sister ah ......" "You are really taking a knife and ruthlessly into my heart ah! ......"------- --- In this world, it is not the ghosts and gods that are scary, but sometimes it is really the people. | A和B都嫁了人,B是知道A家打理生意的所有事的,B也会向A借钱,就拿B家老公的照片去问医护人员：你们这边最近是不是有这个人在帮忙采购,A的姐姐帮着打理生意十多年,A和B都是普通家庭,A甚至都去B家帮着对方母亲穿寿衣,那会B的老公说是在做点小生意,——————————两年前在A家最苦最难的时候——父母病重,而A和B,B还欠着A一万多,两家人都认识,A本身不是大方的人,B照着A的意见去做,于是B就去问A,不过却在最近遇到了个小麻烦——在采购上一直得不到客户的满意,于是A家姐姐失去了这份珍贵的工作,她姐姐毕竟也曾在医院做了十几年生意,后来B的老公也开始做生意做采购,A和B相识相知,A的姐姐听完,A和姐姐一起去照顾,但结婚不到两年,A听完B的困扰,而B家的生意如日中天,帮着父母打理一家小店铺,借来借去让B家慢慢周转,A的姐姐当机立断,A就发给她姐姐,也是B完完全全知道这些事的时候,A和B关系越来越亲密,下边称A和B,就问了下有没有B家老公的照片,也知道A家从无到有,A家的老父亲身体更加不好,A就彻底沦陷在“被最亲的闺密背叛”和“害苦了自己的姐姐”的自责里走不出来,年轻时她姐姐爱上了一个男人。  A and B are married, B is aware of the A family to take care of business all the things, B will also borrow money from A, will take the B family husband's photo to ask the medical staff: your side recently is not this person in the help of purchasing, A's sister to help take care of business for more than a decade, A and B are ordinary family, A even went to B's family to help each other's mother to wear a birthday suit, that will B's husband said it is doing a little business, the B's husband said. ---------- Two years ago in the A family's most bitter and most difficult time - the parents are seriously ill, and A and B, B still owes A more than 10,000, the two families know each other, A itself is not a generous person, B according to A's advice to do, so B went to ask the A, but but recently encountered a small problem - - in the purchase has not been able to get the customer's money. -In the procurement has not been able to customer satisfaction, so the A sister lost this precious job, her sister, after all, had also done business in the hospital for more than ten years, and then B's husband also began to do business to do procurement, A and B know each other, A's sister listened to, A and his sister to take care of it together, but less than two years of marriage, A listened to B's troubles, and B's business as the day of the week! The first is to help the parents to take care of a small store, borrow to borrow to let the B family slowly turnover, A's sister on the spot, A sent to her sister, but also B fully aware of these things when, A and B relationship is more and more intimate, the next said A and B, asked if there is a B husband's photo, but also know that the A family from scratch, the A family of the old father's health is even more bad, A completely fell into in the "betrayed by the closest girlfriend" and "suffer his sister" of self-blame, her sister fell in love with a man when she was young. |
| 4 | 女人抛夫弃子奔初恋，结局是这样的现实......李婷（化名）是重庆人，当年与初恋被迫分手后，她选择去了广东打工，在学技师期间认识了一个广东男孩。也许是为了尽快让情伤愈合吧，两人认识不到半年，她就和那男人结了婚，还连续生下两个女儿。她有自己经营的店面，生活中丈夫虽然木讷，但对她很好。虽平平淡淡但也平平安安过了十多年。没想到5年前，人到中年的李婷突然接到了初恋方强（化名）的电话。这个电话打破了她的生活，也让她的命运改变了。也许是有初恋情缘的缘故吧，两人很快就聊到了一起。初恋说她每天都想她，找了很多以前的朋友才联系到了她。在随后每天“亲爱的干吗呢”的浪漫问候，和睡前的“宝贝晚安”中，李婷仿佛又回到两人的初恋时，她每天享受在方强带给她的甜言蜜语中。在得知方强想开店做生意，而想经营的正是自己在做事决定帮他。于是她便回了一趟老家见了初恋方强。多年不见两人有聊不完的话，期间方强告诉她自己已离婚多年，一直忘不掉她。在方强的再次强追猛打之下，她沦陷了。“一把年纪了，该为自己活一把了”，李婷将方强的话听进去，也行出来了。回到广州后，她向丈夫提出了离婚，为了尽快回到方强身边，她不惜将大半财产和大女儿留给了丈夫。欢天喜地的李婷飞回到了初恋的身边，准备好好爱一回活一回。两人以夫妻名义开了几年的美容店，这期间方强负责收银，李婷负责接待客户。虽然说话了一人一般分成，但她从来不问进账多少，方强给她多少就多少，从不会主动要求算账什么的。在李婷的心里，觉得两人是一家人，不需要分的那么清。所以即便两人同居了几年，方强始终以“不想影响在读大学的孩子”为借口，不肯与她领结婚证，她也没怨言。不仅如此，方强还会经常回前妻家居住，可对于这些，李婷都不介意。因为方强每天都给她发爱的信息。答应她后半辈子会给她足够保障好好爱她、疼她的。李婷将方强的每一条关爱信息，都宝贝一样的收藏着。然而虚假的东西是经不起摔打的，当去年李婷被查出患有乳腺癌需要化疗和手术时，初恋开始翻脸了。不但赶她走，甚至见都不愿见她，非常的绝情。李婷觉得很委屈，这几年两人开店是以夫妻名义做的，最后方强把钱全部拿走了，连化疗钱都没给她。关键她辛苦经营的店，后来成了初恋与前妻的夫妻店。直到这时她才意识到，从头到尾都是初恋安排的骗局，可惜已经晚了。现在李婷生活很困难了，当时离婚还带回了小女儿需要照顾，不知道接下来该何去何从。对于李婷的遭遇，网友们一边倒的“活该”二字送给她。可怜之人必有可恨之处，为了少女时代的浪漫扔下孩子抛弃了老公。同居两年只是帮他干了两年活，除了信息上的爱，一样没得到。一把岁数的人了，放着好好的日子不过，学年轻人任性。即便不和你领证他怕影响孩子，那为什么既然是合伙做生意，没有合同，又不谈钱呢？一点后路不留，真是够大方！对于爱情，大家一定要保持一定的理性，千万不要被那种不实际的东西蒙蔽了心。眼“盲”了、心可千万不能再瞎了。  Women abandon their husbands and children to run to their first love, the end is such a reality ...... Li Ting (a pseudonym) is a Chongqing people, that year and the first love was forced to break up, she chose to go to Guangdong to work, in the study of technicians during the acquaintance of a Guangdong boy. Perhaps in order to let the love wound heal as soon as possible, the two know less than half a year, she married the man, but also gave birth to two daughters consecutively. She has her own store, life, although her husband mute, but very good to her. Although ordinary but also peacefully over more than ten years. Unexpectedly five years ago, the middle-aged Li Ting suddenly received a phone call from her first love, Fang Qiang (a pseudonym). This phone call broke her life, but also let her fate changed. Perhaps it is because there is a first love affair, the two soon chatted together. First love said she wanted her every day, looking for a lot of former friends to contact her. In the subsequent every day "darling dry it" romantic greetings, and before going to bed in the "baby good night", Li Ting as if back to their first love, she enjoys every day in the Fang Qiang to bring her sweet words. After learning that Fang Qiang wants to open a store to do business, and want to run their own in the work decided to help him. So she went back to her hometown to see her first love Fang Qiang. Years of not seeing the two have endless words, during which Fang Qiang told her that he has been divorced for many years, has not forgotten her. Under Fang Qiang's strong pursuit again, she fell. "At her age, she should live for herself", Li Ting listened to Fang Qiang's words and walked out. After returning to Guangzhou, she filed for divorce from her husband. In order to return to Fang Qiang as soon as possible, she spared most of her property and her eldest daughter to her husband. Joyful Li Ting flew back to the side of the first love, ready to love and live a good time. The two opened a beauty store in the name of husband and wife for a few years, during which time Fang Qiang was responsible for the cash register and Li Ting was responsible for receiving customers. Although the talk of a person generally divided, but she never asked how much incoming, Fang Qiang give her how much on how much, never take the initiative to ask for accounting and so on. In Li Ting's heart, feel that the two are family, do not need to be divided so clear. So even if the two have been living together for several years, Fang Qiang always "do not want to affect the children in college" as an excuse, refused to get a marriage license with her, she did not complain. Not only that, Fang Qiang will often go back to his ex-wife's home to live, but for these, Li Ting do not mind. Because Fang Qiang sent her love messages every day. He promised her that he would give her enough security to love and care for her for the rest of her life. Li Ting treasured every one of Fang Qiang's loving messages like a treasure. However, false things can't stand up to the beating, and when Li Ting was diagnosed with breast cancer last year and needed chemotherapy and surgery, her first love began to flip out. Not only did he drive her away, he didn't even want to see her, he was very desperate. Li Ting felt very aggrieved, these years the two opened the store was done in the name of husband and wife, and finally Fang Qiang took all the money, even chemotherapy money did not give her. The key to her hard work to run the store, and then became the first love and ex-wife's husband and wife store. Only then did she realize that it was a scam arranged by her first love from beginning to end, but unfortunately it was already too late. Now Li Ting life is very difficult, when the divorce also brought back a small daughter to take care of, do not know what to do next. For Li Ting's encounter, netizens one-sided "deserved" word to her. Poor people will be hateful, in order to teenage romance throw down the child abandoned her husband. Cohabitation for two years just to help him do two years of work, in addition to information on the love, the same did not get. A number of years of age, put a good day not, learn young people capricious. Even if you do not get a license he is afraid of affecting the child, then why since it is a partnership business, no contract, and do not talk about money? A little back road does not leave, really generous enough! For love, we must maintain a certain degree of rationality, do not be that kind of impractical things blinded by the heart. Eye "blind", the heart can not be blind again! | 李婷仿佛又回到两人的初恋时,人到中年的李婷突然接到了初恋方强（化名）的电话,两人认识不到半年,李婷都不介意,李婷将方强的每一条关爱信息,这几年两人开店是以夫妻名义做的,所以即便两人同居了几年,期间方强告诉她自己已离婚多年,于是她便回了一趟老家见了初恋方强,欢天喜地的李婷飞回到了初恋的身边,现在李婷生活很困难了,两人以夫妻名义开了几年的美容店,连化疗钱都没给她,同居两年只是帮他干了两年活,多年不见两人有聊不完的话,觉得两人是一家人,最后方强把钱全部拿走了,方强给她多少就多少,李婷觉得很委屈,在得知方强想开店做生意,甚至见都不愿见她,初恋说她每天都想她,两人很快就聊到了一起,李婷将方强的话听进去,因为方强每天都给她发爱的信息,结局是这样的现实......李婷（化名）是重庆人,又不谈钱呢,从头到尾都是初恋安排的骗局,方强还会经常回前妻家居住,后来成了初恋与前妻的夫妻店,千万不要被那种不实际的东西蒙蔽了心,在李婷的心里,即便不和你领证他怕影响孩子,方强始终以“不想影响在读大学的孩子”为借口,在方强的再次强追猛打之下,为了尽快回到方强身边,一把岁数的人了,在学技师期间认识了一个广东男孩,还连续生下两个女儿。  Li Ting as if back to the two first love, people to the middle-aged Li Ting suddenly received the first love Fang Qiang (a pseudonym) of the phone, the two know less than half a year, Li Ting do not mind, Li Ting will be Fang Qiang every care information, the two opened a store in the name of the couple to do this, so even if the two cohabited a few years, during the period of Fang Qiang told her that she has been divorced for many years, so she went back to a trip to his hometown to see his first love Fang Qiang, the first love Fang Qiang, the first love Fang Qiang, the first love Fang Qiang. Joyful Li Ting flew back to the side of the first love, now Li Ting life is very difficult, the two in the name of husband and wife opened a few years of beauty store, even chemotherapy money did not give her, cohabitation for two years just to help him do two years of work, many years of not seeing the two have to talk about the words, feel that the two are a family, and finally Fang Qiang took all the money, Fang Qiang to her how much, Li Ting feel very aggrieved, in the knowledge that the Fangqiang would like to open the store to do business, and even see do not want to meet the first love Fang Qiang. The first love said she wanted to see her every day, the two soon chatted together, Li Ting will Fang Qiang words to listen to, because Fang Qiang every day to send her love messages, the end is such a reality ...... Li Ting (a pseudonym) is Chongqing people, and do not talk about money it, from beginning to end is the first love arranged by the scam, Fang Qiang will often go back to his ex-wife's home to live, and later became the first love and ex-wife's husband and wife store, do not be that kind of impracticality blinded by the heart, in the heart of Li Ting, even if you do not and you do not get a license he was afraid of the impact of the child, Fang Qiang has always been "do not want to influence the children in the university" as an excuse. "as an excuse, in the Fangqiang again strong pursuit under the pounding, in order to return to Fangqiang as soon as possible, a number of years old, in the study of technicians during the acquaintance of a Guangdong boy, but also gave birth to two consecutive daughters. |
| 5 | 一个家庭的败落，有它的渊源。今年是辛丑年，想不到他姐家遇到了三件重大事情，每一件打击感，对整个家族来之振动。我不会对他姐家人以往的总结，但他(她)们所接受今天的惩罚，一定是有原因的，因果是离不开，起的什么因，一定会结的什么果。一月份，他姐的大女儿，检查出乳腺癌晚期，己经转移到肝肾。她退休前是保健院的B超医生，经她手做B超的人有千千万，也不知道帮助查出来了多少癌症患者，维独对自己没有很好重视。她的丈夫又是出名的外科专家，不知道有多少患者，经他初诊，得到了很好的治疗，维独错过了自己妻子的早期诊断，早期治疗。也许是他(她)们都是为别人想大多了，忘记了自己也是一个有血有肉的身体，也需要爱护。姐夫：农历四月初，跌了一跤，左手碗骨折，高血压脑病，现已失去了从前的风采，也失去了做人的遵严，吃喝拉撒都要别人的帮助。退休前是一名堂堂正正的县委副书记，两届县人大主任，退休后是老促会的会长，不知道为全县人民贡献多少自己的光和热。现在落到这个结局，谁也不敢去想。姐夫的儿子，在公安机关工作，为公安侦破各种遇难案件，胜不可数，从一个普通的公安民警，一步一个脚印，达实做好，提任到公安局长。想不到的事情发生了，上月市纪检部门带去后留署，目前为止，得出结论有严重违纪违法行为。姐夫：八十多岁了，他女儿也将近六十，他儿子五十出头。原来是有多少人崇拜的一个家庭地位，现在一年内发生如此难于承受的三件大事，为之痛惜。造成今天的结局，是有原因的，维一不可思量它的根源是家里人，无法想象和理解。国有国法，家有家规，弱爱是一把双刃剑，剌伤他人，也伤害自己。遵守国法家规，然后做一个平常人，一生知足常乐，平安，健康，顺利过好自己，这也是对自己的重要负。  The downfall of a family has its origins. This year is the year of Xinchou, unexpectedly his sister's family encountered three major things, each strike sense, to the whole family to vibrate. I will not summarize his sister's family in the past, but he (she) they received today's punishment, there must be a reason, cause and effect is inseparable, what started what cause, will certainly end what fruit. In January, his sister's eldest daughter was found to have advanced breast cancer, which had metastasized to her liver and kidneys. Before her retirement, she was an ultrasound doctor in a health center. She had done ultrasound on thousands of people and helped find out how many cancer patients, but she did not pay much attention to herself. Her husband is a famous surgical expert, I don't know how many patients, after his initial diagnosis, got good treatment, Widu missed his wife's early diagnosis, early treatment. Maybe he (she) are for others think mostly, forgetting that they are also a flesh and blood body, also need to love and care. Brother-in-law: early April of the lunar calendar, fell, left hand bowl fracture, hypertensive encephalopathy, has lost the former elegance, but also lost the human compliance, eating, drinking, and have to be helped by others. Before retirement is a hallowed deputy secretary of the county party committee, two county people's congress director, after retirement is the old promote the president of the association, don't know how much for the people of the county to contribute their own light and heat. Now fall to this end, who do not dare to think. Brother-in-law's son, working in the public security organs, for public security to solve all kinds of distress cases, win uncountable, from an ordinary public security police, step by step, up to real good, appointed to the public security bureau chief. Unexpected things happened, last month, the city discipline inspection department brought to stay after the Department, so far, concluded that there are serious disciplinary violations. Brother-in-law: more than eighty years old, his daughter is nearly sixty, his son in his early fifties. The original is how many people worship a family status, now within a year so difficult to bear the three major events, deplored. Caused today's end, there is a reason, Viichi unthinkable it is the root cause of the family, can not imagine and understand. State law, family rules, weak love is a double-edged sword, stabbing others, but also hurt themselves. Compliance with state law and family rules, and then do a normal person, a life of contentment, peace, health, and smooth over their own, which is also an important responsibility for their own. | 我不会对他姐家人以往的总结,想不到他姐家遇到了三件重大事情,得到了很好的治疗,姐夫的儿子,从一个普通的公安民警,不知道有多少患者,然后做一个平常人,一个家庭的败落,原来是有多少人崇拜的一个家庭地位,也不知道帮助查出来了多少癌症患者,谁也不敢去想,她退休前是保健院的B超医生,想不到的事情发生了,他姐的大女儿,维独对自己没有很好重视,因果是离不开,现在落到这个结局,早期治疗,经她手做B超的人有千千万,一定是有原因的,是有原因的,家有家规,也许是他(她)们都是为别人想大多了,姐夫：八十多岁了,姐夫：农历四月初,维独错过了自己妻子的早期诊断,顺利过好自己,现在一年内发生如此难于承受的三件大事,忘记了自己也是一个有血有肉的身体,不知道为全县人民贡献多少自己的光和热,退休前是一名堂堂正正的县委副书记,吃喝拉撒都要别人的帮助,在公安机关工作,造成今天的结局,为公安侦破各种遇难案件,遵守国法家规,他儿子五十出头,退休后是老促会的会长,他女儿也将近六十,每一件打击感,维一不可思量它的根源是家里人,一定会结的什么果,有它的渊源,今年是辛丑年,对整个家族来之振动,但他(她)们所接受今天的惩罚,起的什么因,一月份。  I will not summarize his sister's family in the past, I can not imagine his sister's family encountered three major things, got good treatment, brother-in-law's son, from an ordinary public security police, I do not know how many patients, and then do a normal person, a family's downfall, the original is how many people worship the status of a family, and I do not know how many cancer patients to help find out who dared not think that she retired before the health center ultrasound doctor, I do not know how many patients, but I can not think of a family of the family. Health center ultrasound doctor, unexpected things happened, his sister's eldest daughter, Vidocq did not pay very good attention to themselves, cause and effect is inseparable, and now fall to this end, early treatment, through her hands to do ultrasound people have thousands of people, there must be a reason, there are reasons, there are reasons, there are rules of the family, maybe it is he (she) are for others to think about the majority of the brother-in-law: more than eighty years old, sister-in-law: the beginning of the fourth month of the lunar calendar, Vidocq. Missed his wife's early diagnosis, smooth over their own, and now so difficult to bear the three major events within a year, forgetting that he is also a flesh and blood body, do not know how much for the people of the county to contribute their own light and heat, retired before the county party committee is a dignified deputy secretary, eating, drinking and drinking are to be other people's help, in the public security organs, resulting in today's end, for the public security to detect a variety of distress The case,abercrombie france, abide by national laws and family rules, his son is in his early fifties, retired, is the president of the old promote the Association, his daughter is also nearly sixty, every hit feeling, Wei Yi can not think of its root is the family, will certainly be the fruit of what has its origin, this year is the year of the xin chou, the vibration of the whole family to come, but he (she) they accepted today's punishment, the beginning of the cause of what, January, the first year of the year, the first year of the first year of the second year of the first year of the first year of the first year of the first year of the first year of the first year of the first year of the first month. |
