# Supplementary material for: Differing Content and Language Based on Poster-Patient Relationships on the Chinese Social Media Platform Weibo: Text Classification, Sentiment Analysis, and Topic Modeling of Posts on Breast Cancer
Source: JMIR Cancer. 2024 May 9;10:e51332. doi: 10.2196/51332 (PMC11117131; doi:10.2196/51332)
Supplement: Multimedia Appendix 3 [file cancer_v10i1e51332_app3.docx]

**Table S2.** Top 30 terms of top 30 topics from topic modeling.

| ID | Topic number | The summarized theme | Top 30 representative words (Chinese) | Top 30 representative words (translated into English) |
| --- | --- | --- | --- | --- |
| 0 | 0 | Anger | 生气,会得,脾气,气死我了,情绪,真的,乳腺癌,发脾气,每次,不想,生闷气,发泄,吵架,气出,玉兔,事情,子宫癌,讨厌,控制,憋着,不好,气死,灯火,身体,天天,发火,宫颈癌,告诉,莫生气,说话 | angry, will get, temper, angry me, emotions, really, breast cancer, tantrum, every, don't want, sulk, vent, quarrel, gas out, jade rabbit, things, uterine cancer, hate, control, hold, bad, gas out, lights, body, every day, fuming, cervical cancer, tell, don't get angry, talk |
| 1 | 1 | Laments | 去世,家里,回来,生活,记得,几天,两个,电话,离开,回家,好像,晚期,事情,昨天,时间,消息,打电话,乳腺癌,眼泪,去年,身边,几年,死亡,听说,生命,吃饭,小时候,真的,年轻,痛苦 | passed away, home, back, life, remember, days, two, phone, leave, home, as if, late, thing, yesterday, time, news, call, breast cancer, tears, last year, around, years, death, heard, life, eating, as a child, really, young, pain |
| 2 | 3 | Symptoms | 乳腺,乳房,肿块,增生,结节,淋巴结,检查,手术,切除,肿瘤,腋窝,医生,疼痛,肿大,皮肤,乳腺癌,乳头,超声,患者,早期,转移,良性,淋巴,医院,情况,化疗,ct,全切,pet,治疗 | breast, breast, lump, hyperplasia, node, lymph node, examination, surgery, excision, tumor, armpit, doctor, pain, enlargement, skin, breast cancer, nipple, ultrasound, patient, early, metastasis, benign, lymph, hospital, condition, chemotherapy, ct, total excision, pet, treatment |
| 3 | 4 | Hospital stays | 医生,病人,主任,医院,手术,换药,检查,下午,患者,大夫,输液,出院,明天,住院,化疗,治疗,查房,老人,手术室,小时,科室,做手术,紧张,回家,伤口,复查,两个,昨天,病房,早上 | doctor, patient, director, hospital, surgery, change, check, afternoon, patient, doctor, infusion, discharge, tomorrow, hospitalization, chemotherapy, treatment, check, elderly, operating room, hours, department, do surgery, nervous, home, wound, review, two, yesterday, ward, morning |
| 4 | 7 | Hope and prayers | 希望,幸福,生活,人生,幸运,健康,生命,经历,活着,世界,勇敢,坚强,乐观,祈祷,真的,珍惜,身体,努力,快乐,痛苦,面对,朋友,生病,科比,乳腺癌,事情,癌症,鼓励,年轻,接受 | hope, happiness, life, life, lucky, health, life, experience, live, world, brave, strong, optimistic, pray, really, cherish, body, try, happy, pain, face, friend, sick, Kirby, breast cancer, things, cancer, encourage, young, accept |
| 5 | 6 | Hospitalization | 手术,医院,化疗,住院,医生,治疗,穿刺,检查,术后,切除,复查,转移,确诊,病房,10,社保,复发,一年,患者,建议,报销,疫情,病理,乳腺癌,恢复,乳腺,乳房,肿瘤,放疗,经历 | surgery, hospital, chemotherapy, hospitalization, doctor, treatment, puncture, examination, postoperative, excision, review, metastasis, confirmed, ward, 10, social security, recurrence, year, patient, advice, reimbursement, epidemic, pathology, breast cancer, recovery, breast, breast, tumor, radiotherapy, experience |
| 6 | 8 | Lamenting hospitalization | 病房,医院,病人,恐惧,患者,住院,看着,隔壁,化疗,真的,治疗,死亡,旁边,手术,病友,晚期,痛苦,医生,放弃,乳腺癌,身体,生命,眼睛,转移,一句,聊天,说话,一位,希望,两位 | ward, hospital, patient, fear, patient, hospitalization, watch, next door, chemotherapy, really, treatment, death, next to, surgery, patient, advanced, pain, doctor, give up, breast cancer, body, life, eyes, metastasis, sentence, chat, talk, one, hope, two |
| 7 | 2 | Dreams and nightmares | 梦里,梦见,梦到,昨晚,做梦,醒来,噩梦,抢救,好像,真实,乳腺癌,昨天,惊醒,晚上,折腾,昨天晚上,手机,真的,癌症,医院,吓醒,病人,早上,床上,小时,闹钟,一场,睡觉,哭醒,发生 | dream, dreaming, dreaming, last night, dreaming, waking, nightmare, resuscitation, as if, real, breast cancer, yesterday, woke up, night, tossed, last night, phone, really, cancer, hospital, woke up, patient, morning, bed, hour, alarm clock, a, sleep, woke up crying, happened |
| 8 | 10 | Diagnosis | 一年,手术,去年,确诊,希望,查出,化疗,健康,身体,乳腺癌,年前,我要,治疗,癌症,真的,生活,查出来,看着,转移,五年,告诉,检查,扩散,努力,我会,生病,见到,加油,时间,接受 | a year, surgery, last year, diagnosed, hope, find out, chemo, health, body, breast cancer, years ago, I want, treatment, cancer, really, life, find out, look, metastasis, five years, tell, check, spread, try, I will, sick, see, cheer, time, accept |
| 9 | 5 | Chinese dramas | 刘静,女主,男主,欢喜,英子,方圆,剧情,这部,宋倩,编剧,童文洁,导演,黄磊,影片,角色,韦一航,演员,电影,结局,电视剧,嫉妒,爱情,剧中,感情,季杨,真的,方一凡,三少,马小远,生活 | Liu Jing, heroine, hero, cheerful, Yingzi, Fang Yuan, plot, this, Song Qian, screenplay, Tong Wenjie, director, Huang Lei, film, role, Wei Yihang, actor, movie, ending, drama, jealousy, love, drama, feelings, Ji Yang, really, Fang Yifan, San Shao, Ma Xiaoyuan, life |
| 10 | 13 | School | 老师,学生,家长,班主任,上课,初中,学校,教师,小学,高中,教育,作业,真的,学习,班级,班上,记得,全班,教室,英语老师,数学,学期,寒暑假,工资,小朋友,毕业,下课,语文,乳腺癌,职业 | teacher, student, parent, classroom, lesson, middle school, school, teacher, elementary, high school, education, homework, really, study, class, class, class, remember, whole class, classroom, english teacher, math, semester, summer and winter, salary, kids, graduation, off, class, language, breast cancer, career |
| 11 | 20 | Friends | 朋友,闺蜜,离婚,聊天,命理,人生,事情,乳腺癌,大学,未必,对象,话题,生活,赵姐,散步,查出,一位,刚刚,可惜,完整,身边,李女士,真的,希望,发生,谁谁谁,能力,条件,糟糕,不想 | friend, bestie, divorce, chat, numerology, life, thing, breast cancer, college, may not, object, topic, life, Zhao, walk, find out, one, just, unfortunately, complete, around, Ms. Li, really, hope, happen, who, who, ability, condition, bad, do not want |
| 12 | 18 | Sleep-wake cycles | 熬夜,睡觉,晚上,睡不着,睡着,晚睡,中午,手机,睡眠,白天,凌晨,天天,小时,舒服,吃饭,内分泌,早上,十二点,关灯,乳腺癌,李姐,每晚,时间,厕所,失眠,头痛,真的,不好,身体,几天 | stay up, sleep, night, sleepless, sleep, late, noon, phone, sleep, day, early morning, daily, hours, comfortable, eating, endocrine, morning, twelve o'clock, lights off, breast cancer, Li, night, time, toilet, insomnia, headache, really, bad, body, days |
| 13 | 12 | Passing | 去世,消息,难过,死者,刚刚,告诉,去年,乳腺癌,得知,离开,听说,一位,难受,当年,生命,怀着,省钱,查出,心疼,小丸子,真的,意外,想起,姚贝娜,过世,拼命,胡歌,遗憾,两个,小时候 | passed away, news, sad, deceased, just, told, last year, breast cancer, learned, left, heard, one, hard, back then, life, pregnant, save money, found out, heartache, small pill, really, unexpected, remember, Yao Beina, passed away, desperately, Hu Ge, regret, two, as a child |
| 14 | 26 | Treatment processes | 放疗,化疗,结束,治疗,转移,复查,建议,医生,淋巴结,药物,方案,免疫组化,内分泌,免疫治疗,病理,淋巴,医院,三阴,手术,临床试验,副作用,白细胞,肿瘤,靶向,确诊,检查,主任,希望,娜娜,腋下 | radiotherapy, chemotherapy, end, treatment, metastasis, review, advice, doctor, lymph node, drug, protocol, immunohistochemistry, endocrine, immunotherapy, pathology, lymph, hospital, triple negative, surgery, clinical trial, side effects, white blood cell, tumor, targeted, confirmed, examination, director, hope, nana, armpit |
| 15 | 33 | Treatment effects | 治愈,治疗,方案,效果,患者,类型,阳性,分型,复发,化疗,靶向,转移,三阴,预后,内分泌,术后,乳腺癌,中医,药物,情况,医书,不好,医生,晚期,病情,国产,吃药,进口,比星,癌症 | cure, treatment, protocol, effect, patient, type, positive, fraction, recurrence, chemotherapy, targeted, metastasis, triple negative, prognosis, endocrine, postoperative, breast cancer, TCM, drug, situation, medical book, bad, doctor, late, condition, domestic, take, import, bixin, cancer |
| 16 | 113 | Appeal to emotion | 开心,心情,事情,几率,难过,郁结,我要,生闷气,活着,乳腺癌,cp,情绪,不想,真的,增加,会得,身体健康,样子,pua,希望,快乐,晚上,于心,冰淇淋,白头发,官姐,浪漫,不快,而活,抑郁 | happy, mood, things, odds, sad, depressed, I want, sulking, alive, breast cancer, cp, emotions, don't want, really, increase, will get, physical health, look, pua, hope, happy, night, in the heart, ice cream, white hair, official sister, romantic, unhappy, while living, depression |
| 17 | 42 | Initiative | 面对,压力,生活,健康,人生,努力,乐观,想要,未来,真的,长大,经历,痛苦,希望,得知,快乐,真心,真实,艺术,短暂,乳腺癌,子女,妇科,一期,几年,接受,倒计时,想到,办法,往后 | face, pressure, life, health, life, effort, optimism, want, future, really, grow up, experience, pain, hope, learn, happy, sincere, true, art, brief, breast cancer, children, gynecology, phase, years, accept, countdown, think, way, backward |
| 18 | 11 | A Little Red Flower (A popular Chinese movie released in 2020) | 小花,一朵,千惠,小红花,病魔,崔雅,悲伤,生活,日子,生命,stephen,命运,经历,意外,平安,希望,抗癌,纯辉,平凡,电影,绝望,美少女,离开,一颗,永远,朋友,乳腺癌,不幸,坚强,劳拉 | little flower, a, Chie, little red flower, disease, cuiya, sad, life, days, life, stephen, fate, experience, accident, peace, hope, fight cancer, pure fai, ordinary, movie, despair, beautiful girl, leave, a, forever, friend, breast cancer, misfortune, strong, Laura |
| 19 | 45 | Suspicion of breast cancer | 怀疑,焦虑症,返祖,胸痛,检查,担心,大肠癌,血细胞,怕死,百度,癌胚抗原,肠胃,胃癌,焦虑,组长,医院,脖子,乳腺,心理,症状,雪君,高度,癌症,乳腺癌,指标,一度,寂寞,走路,人生,含量 | suspicion, anxiety, revert, chest pain, examination, worry, colon cancer, blood cells, fear of death, baidu, carcinoembryonic antigen, gastrointestinal, stomach cancer, anxiety, group leader, hospital, neck, breast, psychological, symptoms, xuejun, highly, cancer, breast cancer, indicator, once, lonely, walking, l ife, content |
| 20 | 48 | Other cancers | 肺癌,肝癌,胃癌,肠癌,吸烟,抽烟,胰腺癌,癌症,生闷气,朋友,食物,晚期,宫颈癌,柯美,食管癌,乳腺癌,膀胱癌,红肉,发生,可怕,失调,饮食,油腻,喝酒,白血病,基因,西兰花,直肠癌,结肠癌,十天 | lung cancer, liver cancer, stomach cancer, bowel cancer, smoking, smoking, pancreatic cancer, cancer, sulking, friends, food, late, cervical cancer, Comet, esophageal cancer, breast cancer, bladder cancer, red meat, occurrence, terrible, disorder, diet, greasy, drinking, leukemia, genes, broccoli, rectal cancer, colon cancer, ten days |
| 21 | 64 | Anxiety | 焦虑,担心,烦躁,考研,心情,春节,航航,不好,身边,害怕,主流,情绪,放下,真的,能量,生病,公教,抱怨,好多,循环,舒服,乳腺癌,心慌,身体,上班,陷入,看书,事情,更好,子宫 | anxiety, worry, irritable, exam, mood, spring, flight, bad, around, fear, mainstream, emotions, put down, really, energy, sick, public education, complain, much, cycle, comfortable, breast cancer, panic, body, work, caught, reading, things, better, uterus |
| 22 | 17 | Metastasis of cancer cells | 转移,癌症,癌细胞,患者,闫宏微,面对,充满,坚强,治疗,背后,乳腺癌,人间,选择,死亡,希望,仿佛,学会,离开,心中,微微,默默,生活,未曾,播客,想起,疾病,感恩,痛苦,抗癌,病人 | transfer, cancer, cancer cells, patient, Yan Hongwei, face, full, strong, treatment, behind, breast cancer, earth, choice, death, hope, as if, learn, leave, heart, micro, silent, life, untouched, podcast, remember, disease, grateful, pain, fight, cancer, patient |
| 23 | 22 | Weibo follows | 关注,微博,抗癌,荔枝,记录,有人,朋友圈,微博上,更新,惊讶,评论,草药,略略,好久没,捐款,海参,发微博,乐观,世里,文章,胡歌,癌症病人,博主,媒体,乳腺癌,患者,去世,群体,经历,希望 | concern, microblogging, anti-cancer, lychee, record, someone, circle of friends, on microblogging, update, surprised, comment, herbs, slightly, for a long time, donations, sea cucumber, tweeting, optimism, Shili, article, Hu Ge, cancer patients, bloggers, media, breast cancer, patients, died, group, experience, hope |
| 24 | 23 | Weibo usage | 微博,媽媽,努力做到,更新,不想,天天,战哥,真的,微博上,喜歡,客戶,生活,微博是,视频,去世,讨厌,发泄,希望,接受,乳腺癌,胃病,家产,情绪,安葬费,山镇,声音,特別,不诚实,棋盘,政府 | microblogging, mom, trying to do, update, don't want, everyday, war brother, really, on microblogging, like, customer, life, microblogging is, video, passed away, hate, vent, hope, accept, breast cancer, stomach disease, family property, emotions, burial expenses, mountain town, voice, special, dishonest, chessboard, government |
| 25 | 85 | Side-effect of treatments | 头发,假发,化疗,光头,掉头发,睫毛,帽子,剪短,脱发,长发,洗头,眉毛,几根,剃光,第二次,脱落,头上,一顶,一根,打针,一大,焦距,放疗,露出,短发,漂亮,接受,真发,镜子,出门 | hair, wig, chemotherapy, bald, lose hair, eyelashes, hat, cut short, hair loss, long hair, wash, eyebrows, a few, shaved, second, off, head, a top, a, shot, a large, focal, radiotherapy, reveal, short hair, pretty, accept, real hair, mirror, go out |
| 26 | 27 | Check-up | 姐夫,电话,昨天,医生,回去,舒服,医院,雾草,报告,心情,接到,周四,礼拜,体检,两天,胸部,保养,一查,担心,乳腺癌,嘴巴,诊断,放松,难受,一张,检查,三姐,赖子,不好,周五 | brother-in-law, phone, yesterday, doctor, go back, comfortable, hospital, fog grass, report, mood, received, Thursday, week, physical examination, two days, chest, maintenance, a check, worry, breast cancer, mouth, diagnosis, relax, hard, a, check, three sisters, lai, bad, Friday |
| 27 | 63 | Female physiology | 没事,预防,增生,例假,一去,凶宅,流泪,胸就胀,干嘛,调理,已不分,乳腺,医生,尴尬,医院,检查,复查,年龄,心碎,病变,大哭,痛经,三个,擦汗,外痔,乳腺癌,月经,妇科,唤醒,太岁 | Nothing, prevention, hyperplasia, period, a go, murderous house, tears, chest on swelling, why, conditioning, has not divided, breast, doctor, embarrassment, hospital, check, review, age, heartbreak, lesion, cry, dysmenorrhea, three, sweat, external hemorrhoids, breast cancer, menstruation, gynecology, wake, taiyoung |
| 28 | 9 | Public figures | 陈晓旭,李明,伤官,林黛玉,李婷,红楼梦,方强,地支,阿弥陀佛,梁汉文,格局,贫穷,林文慧,郭某,广州,大运,小明,裴某,两人,往生,深圳,心仪,流下来,念佛,真相,角色,校长,宝咏琴,老李,财格 | Chen Xiaoxu, li ming, hurt official, lindaiyu, li ting, hongloumeng, fang qiang, earth branch, amitabha, liang hanwen, pattern, poverty, lin wenhui, guo mou, guangzhou, big luck, xiao ming, pei mou, two people, reincarnation, shenzhen, xingyi, flow down, chanting, truth, role, principal, po wing qin, old lee, wealth pattern |
| 29 | 58 | Treatment stages | 化疗,第二次,第三次,结束,白细胞,胃口,一疗,四次,食道,骨头,紫杉醇,第四次,长效,三疗,打断,脂质体,升白针,回弹,白针,掉头发,关节,两天,舒服,损伤,副作用,比星,打升,四疗,溃疡,心情 | chemotherapy, second, third, end, white cells, appetite, one treatment, four, esophagus, bone, paclitaxel, fourth, long-acting, three treatments, interrupt, liposome, ascending injection, rebound, white injection, hair loss, joints, two days, comfortable, injury, side effects, paclitaxel, playing ascending, four treatments, ulcer, mood |
