# Supplementary material for: Differing Content and Language Based on Poster-Patient Relationships on the Chinese Social Media Platform Weibo: Text Classification, Sentiment Analysis, and Topic Modeling of Posts on Breast Cancer
Source: JMIR Cancer. 2024 May 9;10:e51332. doi: 10.2196/51332 (PMC11117131; doi:10.2196/51332)
Supplement: Multimedia Appendix 4 [file cancer_v10i1e51332_app4.docx]

**Table S3.** Examples of error analysis.

|  | True label | Predicted label | Post |
| --- | --- | --- | --- |
| **Post I** | post_user | acquaintances | The sense of boundaries between people is really too important, at least for me. Today after the meeting a **colleague** stopped by to take me back and chatted about whether my mom would be worried if I lived alone. I didn't talk for two sentences before I jumped to: Is your mom still pushing for marriage (**meaning after having breast cancer**)? Are you planning to find a boyfriend? I thought I was smart enough to say that I had been abandoned because I was sick. The result was that people were able to continue to ask: Is the other party now married? I really confused on the spot, I really want to return a ``none of your business'' or ``none of my business'', if people do not get married for me, from the beginning will not abandon me this is not difficult to understand it? Since they say abandoned me he married or not I have to care? Do not tired? What's wrong with living happily? Must be a pain in the ass? Oh yes, this time it is a woman. |
| **Post II** | post_user | family_members | The unforgettable eleven days is my baby in the hospital to accompany me 11 days, who can not believe that the **father** to replace her to let her go to work can not. **I had breast cancer**, but luckily I have a good **daughter** who is filial, and I also have a good **son** and **daughter-in-law**, they did not come to accompany me because it is not convenient to come during the epidemic, my **granddaughter** is 13 years old and still has to study. When the video knew that **grandma** was sick, she cried and said, ``Why didn't you tell me? Thinking about all this I must be strong to overcome the disease. |
